# Interfacing metal organic frameworks with polymers or carbon-based materials: from simple to hierarchical porous and nanostructured composites

**DOI:** 10.1039/d3sc03659f

**Published:** 2023-10-19

**Authors:** Khaled Dassouki, Sanchari Dasgupta, Eddy Dumas, Nathalie Steunou

**Affiliations:** a Institut Lavoisier de Versailles, UMR CNRS 8180, Université de Versailles St Quentin en Yvelines, Université Paris Saclay Versailles France nathalie.steunou@uvsq.fr

## Abstract

In the past few years, metal organic frameworks (MOFs) have been assembled with (bio)polymers and a series of carbon-based materials (graphene, graphene oxide, carbon nanotubes, carbon quantum dots, *etc*.) leading to a wide range of composites differing in their chemical composition, pore structure and functionality. The objective was mainly to overcome the limitations of MOFs in terms of mechanical properties, chemical stability and processability while imparting novel functionality (electron conductivity, (photo)catalytic activity, *etc*.) and hierarchical porosity. These composites were considered for numerous applications including gas/liquid adsorption and separation, (photo)catalysis, biomedicine, energy storage, conversion and so on. The performance of such composites depends strongly on their microstructural and physico-chemical properties which are mainly driven by the chemical strategies used to design and process such composites. In this perspective article, we propose to cover this topic and provide a useful survey of recent progress in the synthesis and design of MOFs–carbon material composites. This article will describe the development of composites with increasing complexity in terms of porous architecture, spatial structuration and organisation, and functionality.

## Abstract 2

The article covers the survey of recent progress in the design of MOFs–polymers and MOF–carbon material composites with increasing complexity in terms of porous architecture, spatial structuration, organisation, and functionality.

## Graphical abstract

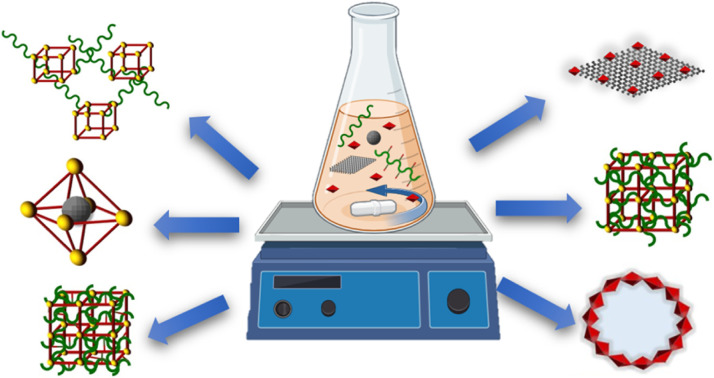

## Introduction

In the past few decades, one of the most prominent areas of research in materials chemistry has been the exploration of novel bio-inspired strategies to prepare inorganic and hybrid materials with controlled sizes, morphologies and defined texture.^1,2^ In particular, there has been significant interest in drawing inspiration from nature to create superstructures that resemble naturally occurring biominerals, with their extraordinary shapes and intricate compositions. Indeed, numerous biominerals such as pearls, sea urchin spines, oyster shells or corals are well-known inorganic/organic composites and one of their remarkable features lies in their hierarchical ordered structure spanning from the nano- to macroscopic scale.^1,2^ Rational synthesis methods based on the co-assembly of inorganic colloids (metals, oxides, metal sulphides, *etc*.) with self-assembled organic superstructures or templates were explored to prepare composite materials with tailored and complex morphology, high porosity and hierarchy. The design of such composites with a high level of morphological complexity, hierarchical dimensionality and porosity, was relevant to the development of novel types of heterogeneous catalysts, separation membranes, biomedical implants or drug delivery nanocarriers.^1,2^

**Fig. 1:**
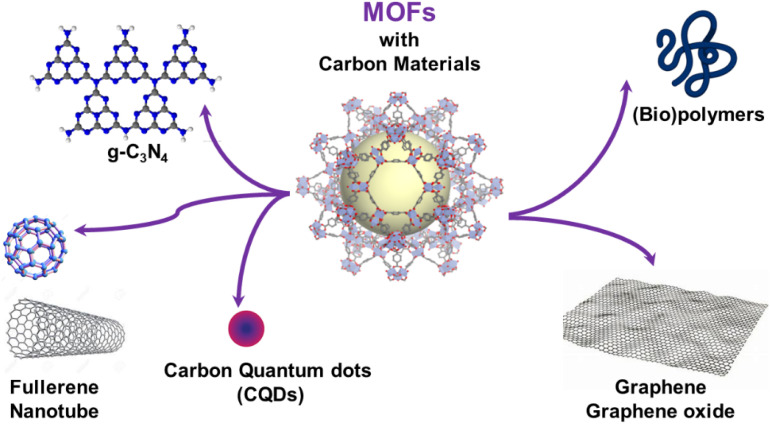
Schematic illustration of the carbon-based materials and (bio)polymers used for the processing of MOF-based composites.

In the continuous quest for novel multifunctional composites, such strategies were expanded to the combination of organic materials with metal–organic frameworks (MOFs). MOFs are a class of porous and crystalline hybrid materials constructed by assembling metal-based nodes and organic polydentate ligands.^3,4^ Their highly ordered pore structures, large surface areas and chemical functionalities make these materials highly promising for a wide range of applications, including gas storage and separation, catalysis, sensing, energy storage and biomedicine among others.^5–9^ However, MOFs still face certain limitations that greatly hinder their transfer and implementation in real industrial applications. A large number of MOFs exhibit limited chemical and thermal stability and thus a poor resistance to water. This can result in the irreversible degradation of their frameworks when exposed to humid air. Additionally, conventional MOF synthesis methods often produce polydisperse powder samples. The low mechanical properties of MOF powders, along with their poor processability and handling properties as well as their low mass/heat transfer rate significantly impede the development of MOF-based applications.^10^ As an example, MOFs have been mainly processed in the forms of pellets, granules, beads, fibres and membranes but in many cases, the accessibility to the MOF porosity is lost after shaping.^10^ A high pressure drop can also be observed for densely packed MOF powders within an adsorption column.^10^ In catalysis, the use of MOF powders hampers the catalyst recycling. To address these issues, various strategies have been explored, primarily involving the integration of MOFs with different supporting materials, such as (bio)polymers or carbon-based materials (carbon nanotubes, graphene, graphene oxide, carbon quantum dots, *etc.*) (Fig. 1).^11–13^ This assembly between both components allows for the creation of multifunctional composites that combine the unique properties of MOFs (porosity and crystallinity) with the desirable characteristics of the supporting matrix (mechanical stability, processability, *etc.*). Moreover, this approach can also enhance the stability, impart electron conductivity, and extend the porosity of MOFs in the meso- or macroporous regime.^11–16^ The hybridization of MOFs with carbon-based materials has resulted so far in a wide range of composites that have found applications in different fields including gas adsorption and separation, sensing, water purification, catalysis, and biotechnology.^11–16^ The high performance of such composites in those applications is required to optimise the MOF loading and the accessible porosity while limiting the presence of interfacial defects. The physico-chemical properties of such composites are thus strongly driven by the control over the spatial distribution and compartmentalisation of MOFs within organic matrices and the MOF/carbon material interfaces. This has been extensively described in the processing of MOF–polymer membranes.^11,12^ Therefore, the microstructural properties of such composites greatly depend on how the components are spatially integrated, and this is determined by the synthetic strategies employed to construct such MOF-based composites.

This perspective article aims to describe recent developments in the design of composites combining MOFs with (bio)polymers or carbon materials (carbon dots, graphene, graphene oxides, *etc*.). Our contribution is not intended to focus on the performance of MOF-based composites in a certain field of application (catalysis, separation, and energy) as it was extensively reviewed elsewhere.^17–21^ In this perspective, we aim to provide an overview and critical discussion of the different synthetic strategies that were deployed for the fabrication of MOF–polymer and MOF–carbon material composites. These synthesis approaches will be divided into five parts, taking advantage of the structural and physico-chemical properties of MOFs including their high porosity, functionality of the framework and the surface chemistry of the particles. The first two parts will be dedicated to composites prepared through the insertion or formation of the carbon-based component either in the pores or in the framework of MOFs. Then, the third and fourth parts will describe the integration of preformed MOFs into organic matrices without altering the MOF porosity. The surface engineering of MOF is the key point of this approach and will be outlined in the third section. The fifth part deals with the design of hierarchically porous MOF/carbon material composites. Note that the cohesion and stability of such composites strongly depend on their interfacial properties. Composites with MOFs incorporated in organic matrices or encapsulating carbon entities are mainly based on electrostatic, van der Waals interactions or hydrogen bonds. However, polymers could also be integrated into the MOF framework through coordination bonds or covalently grafted at the MOF surface. This perspective also aims to emphasise that the design of highly performing composites requires control over the MOF particle size and morphology, the nanostructuration and spatial organisation of components at different length scales as well as the hierarchical porosity in the micro-, meso- and/or macroporous regimes. Thus, this article intends to address the main advances achieved in the design of such hierarchically porous MOF–carbon material composites in the fourth and fifth sections. We expect that this review will provide synthetic guidance for developing the next generation of advanced MOF-based composites.

## Encapsulation of carbon-based nanoentities in the pores of MOFs

**Fig. 2:**
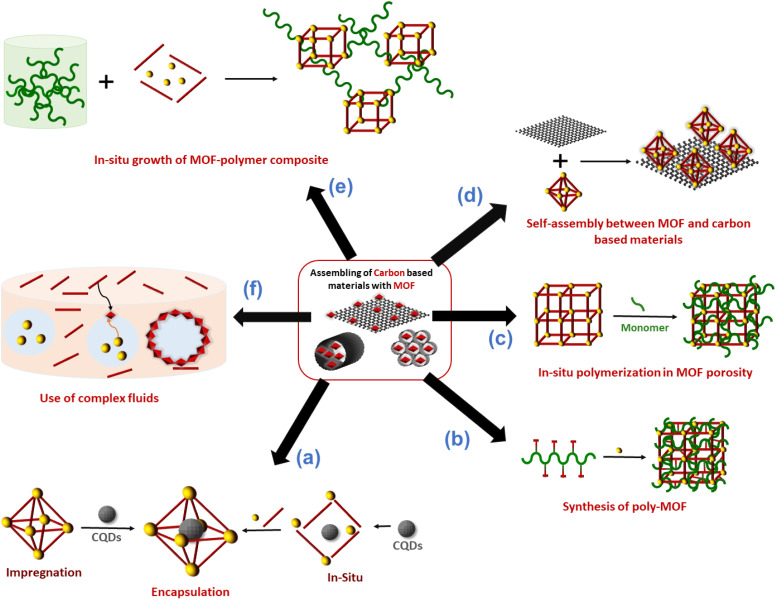
Schematic representation of the different strategies reported in the literature to combine MOFs with carbon materials.

Carbon nanoentities (CNEs) such as fullerene, carbon quantum dots or graphene quantum dots were encapsulated in the pores of MOFs. This approach aims to enhance the chemical stability of CNEs and prevent their aggregation which is detrimental to their physicochemical properties (catalytic and luminescence).^11,22^ The host MOF materials can provide an accessible porosity and also additional active sites, leading to porous composite materials with a synergistic combination of the properties of MOFs and CNEs.^11,22^ The assembly of CNEs and MOFs was performed following two main strategies. A direct mixing method (also referred to as the ship-in-bottle or impregnation strategy) is a stepwise synthesis approach that consists of synthesising separately the CNEs and MOFs followed by their mixing in solution at room temperature (RT)^11,23,24^ (Fig. 2a). This method has the advantages of being simple and rapid but it does not allow a precise location of CNEs within the MOFs since CNEs are inevitably deposited at the surface of MOF particles and/or inserted in the MOF pores. Note that the encapsulation of CNEs in the MOF pores is restricted to mesoporous MOFs whose pore diameters are compatible with the dimension of CNEs and it also relies on the diffusion of CNEs through MOF windows that are smaller than the pore itself. Therefore, for the vast number of microporous MOFs, this method generally leads to the surface decoration of MOF particles by CNEs. This method may also suffer from a high aggregation of CNEs in solution that hinders a homogeneous spatial distribution of CNEs within the MOF crystals.^11,23,24^ To overcome the limitations of the ship-in-bottle strategy, numerous MOF-CNEs were also synthesized by an *in situ* approach (also referred to as the bottle-around-ship strategy) which involved the concomitant nucleation and growth of MOFs with the insertion of CNEs (Fig. 2a).^11,23,24^ This method consists of mixing the MOF precursors (ligands and the metal source) with preformed CNEs and then the synthetic protocol is carried out under the same conditions as for the pure MOF synthesis. This method is not restrained by the MOF porosity and similarly to the ship-in-bottle approach, it allows the entrapment and stabilization of CNEs in a 3D assembly of MOF crystals. The presence of functional groups (carboxylate, hydroxy, and amino) at the surface of CNEs can also act as nucleation sites of the MOFs thereby leading to a high spatial distribution of CNEs within the composite.^3,11,24^ However, in some cases, these capping groups can also compete with the organic ligands for metal coordination, thereby affecting the nucleation and growth of the MOFs. Each strategy, either the ship-in-bottle or bottle-around-ship strategy has its advantages and limitations and was thus selected depending on the MOF/CNE system.

**Fig. 3:**
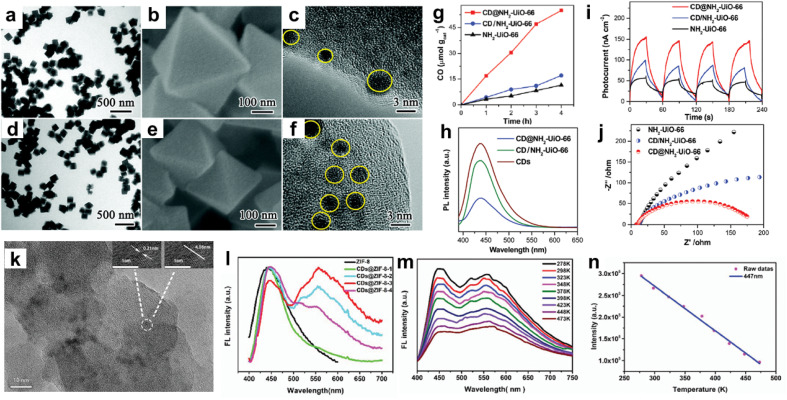
MOFs–CQD composites prepared through different strategies, namely (a–j) ship-in-bottle and bottle-around-ship, and (k–n) thermal post-synthetic treatment of MOFs loaded with organic molecules. (a–f) TEM and SEM images of (a–c) CQD-decorated UiO-66(Zr)-NH_2_ and (d–f) CQD-embedded UiO-66(Zr)-NH_2_, (g–j) characterization of the photocatalytic performance of CQD-embedded UiO-66(Zr)-NH_2_ (*i.e.* CD@NH_2_-UiO-66) in comparison to CQD decorated UiO-66(Zr)-NH_2_ (*i.e.* CD/NH_2_-UiO-66) and pristine UiO-66(Zr)-NH_2_ by coupling measurements of (g) the photocatalytic activity for CO_2_ reduction, (h) photoluminescence (PL) spectra (i) photocurrents and (j) electrochemical impedance spectra (EIS) (adapted with permission from ref. 30 Copyright 2020, Royal Society of Chemistry). (k) TEM image of CQD@ZIF-8, (l) PL spectra of a series of CQD@ZIF-8 compounds with different CQD loadings, (m) temperature-responsive PL spectra of CQD@ZIF-8, and (n) linear correlation of fluorescence intensity with temperature of CQD@ZIF-8 (emission at 447 nm) (adapted with permission from ref. 38 Copyright 2018, Royal Society of Chemistry).

Among CNEs, carbon quantum dots (CQDs) were combined with MOFs and the resulting porous CQDs–MOF composites were mainly investigated for applications in photocatalysis and optical detection.^22,24^ CQDs are a class of carbon nanomaterials with a size lower than 10 nm, consisting of a polyaromatic core stabilised through surface functionalisation.^25^ Their combination of properties such as tunable fluorescence emission, unique photoluminescence stability, good electron conductivity, simple preparation, low cost and good biocompatibility make these nanomaterials very interesting for different fields including sensing, (photo)catalysis and bioimaging.^22,24,25^ In the past few years, a variety of Ti, Zr, Cu or Fe-based MOFs have been explored for photocatalytic application.^5,26,27^ Upon light excitation, the organic linker of the MOF can act as a light absorber and the MOF can undergo a photo-induced charge separation with electrons populating the inorganic building units (IBUs) and holes populating the organic linker.^26,27^ However, their photocatalytic efficiency is yet far from optimal. One major issue is the large band gap of MOFs that restricts their photoresponse to UV light or short-wavelength visible light (<500 nm), thereby largely limiting their applications under the solar spectrum.^26,27^ In addition, most MOF photocatalysts suffer from severe charge recombination.^26,27^ As CQDs can act as both photosensitizers and electron receptors, the combination of MOFs with CQDs was thus explored to improve their photocatalytic efficiency for different reactions such as the degradation of organic pollutants or the CO_2_ reduction reaction (CO_2_RR).^18,24,28,29^ S. Li *et al.* synthesised two types of composites by assembling the porous Zr amino-terephthalate MOF (*i.e.* UiO-66(Zr)-NH_2_) and 3 nm diameter CQDs through either the ship-in-bottle or bottle-around-ship strategy (Fig. 3a–j).^30^ Depending on the synthesis route, these composites were different in terms of microstructure and location of the CQDs. While the ship-in-bottle led to UiO-66(Zr)-NH_2_ nanoparticles (NPs) whose external surface was decorated with CQDs, the *in situ* approach allowed the entrapment of CQDs in the cavities of UiO-66(Zr)-NH_2_ NPs (Fig. 3a–f).^30^ This study aims to investigate the influence of the location of CQD co-catalysts on the photocatalytic activity of UiO-66(Zr)-NH_2_ for the CO_2_RR. It was shown that the CQD-embedded UiO-66(Zr)-NH_2_ photocatalysts outperform both the CQD-decorated UiO-66(Zr)-NH_2_ and the pristine MOF for the CO_2_RR (Fig. 3g–j).^30^ Charge kinetic investigations show that the embedded CQDs significantly improve the charge transfer and separation in comparison to the surface-decorated CQDs due to their spatial proximity with the zirconium-oxo clusters, favouring the formation of numerous heterojunctions (Fig. 3h).^30^ Besides playing the role of electron receptors, CQDs were able to act as photosensitizers once irradiated under long-wavelength light (*λ* > 440 nm).^30^

MOFs have sparked much attention to develop luminescent materials as a result of their inherent luminescent centres located on both the IBU and the organic linker in their framework.^22^ A large number of luminescent MOFs have been reported for applications in sensing, white-light-emitting diodes (LEDs) and biomedicine.^22,31–33^ In particular, a range of MOFs was developed as luminescent detection platforms for various target analyses, such as ions, organic molecules, gases, explosives, humidity, pH and temperature.^22,31–33^ These materials have thus shown great promise for the construction of luminescent sensors with a high selectivity and sensitivity. In addition to the luminescence of the MOF itself, various luminescent guests (lanthanide ions, dyes, luminescent complexes, *etc*.) can also be encapsulated in the MOF pores to expand their photonic functionality.^22^ Among luminescent guests, CQDs present a high photonic quantum yield and high photoluminescence stability that were exploited for the building up of fluorescent probes and sensors.^22^ However, as reported for fluorescent dyes, they easily aggregate in solution and this results in fluorescence quenching.^34,35^ The dispersion of CQDs within MOF matrices was thus explored to avoid this fluorescence aggregation quenching, regulate the fluorescent properties of CQDs or potentially synergistically combine the fluorescent properties of both MOFs and CQDs through adequate host–guest interactions.^22^ Ma *et al.* and J. Yang *et al.* prepared a fluorescence sensor by the *in situ* method, allowing the confinement of CQDs in the cavities of the zeolitic imidazolate framework ZIF-8(Zn).^35,36^ This CQDs@ZIF-8 that combined the yellow fluorescence of CQDs with the blue fluorescence of ZIF-8 took advantage of this dual-emission and was thus used for the ratiometric fluorescence detection of Cu^2+^.^35^ A high diversity of dual-emission fluorescent probes was prepared similarly by the *in situ* method enabling the encapsulation of luminescent guests (preformed CQDs and lanthanide ions) into the MOF pores during the MOF crystallization.^22^ Recently, another approach took advantage of the good thermal stability of a few MOFs. CQDs were formed through the thermal post-synthetic treatment of MOFs loaded with organic molecules (glucose).^37^ This strategy was extended to the non-activated ZIF-8 containing an excess of free imidazolate and its thermal treatment led to the growth of 4 nm sized CQDs in the cavities of ZIF-8 (Fig. 3k–n).^38^ Interestingly, CQDs@ZIF-8 materials are dual-emitting composites exhibiting tunable fluorescence resulting from the inherent blue emission of ZIF-8 and the yellow fluorescence of CQDs whose intensity can vary by controlling the duration of the thermal treatment and thus the amount of CQDs (Fig. 3l).^38^ These composites have shown temperature-responsive photoluminescence properties which opens up their application as temperature sensors (Fig. 3m and n).^38^ It is noteworthy that this thermal post-synthetic treatment of MOFs not only allows tailoring the amount of CQDs but also avoids the concomitant deposition of CQDs at the surface of MOF NPs as it might also occur for the *in situ* strategy. Moreover, MOFs can also provide a powerful confinement effect to limit the growth of CQDs. Therefore, the diameter, stabilisation and optical properties of CQDs are thus driven by specific interactions between CQDs and MOFs.

S.-W. Lo *et al.* took advantage of the host–guest interactions in the pores of MOF-based composites to modulate their optical properties.^39^ More specifically, the buckminsterfullerene (C_60_) was encapsulated in the pores of a chiral MOF by the *in situ* strategy.^39^ Although C_60_ can be described as a highly symmetric truncated icosahedron with the absence of any chirality, the circular dichroism spectrum of the composite showed an intense chiral signal in the absorption region of C_60_.^39^ A series of characterization studies and DFT calculations showed that this unprecedented chiral induction of C_60_ is mediated by strong host–guest interactions, resulting in molecular orbital hybridisation between the chiral MOF and C_60_.^39^ This chiral induction approach was not only of high interest to impart chirality into inherently symmetric molecules but it can also deepen our fundamental knowledge concerning the spontaneous symmetric breaking mechanism.

It is worth noting that numerous composites were similarly prepared through the confinement of a wide range of inorganic and biological nanoentities and such composites have found numerous applications in (bio)catalysis, biomedicine, (bio)sensing and so on.^5,9^ As presented in the next section, polymers could also be encapsulated in the MOF pores while they were also integrated as part of the MOF framework.

## Integration of polymers in the framework or the pores of MOFs

The hybridization of MOFs with polymers has been extensively explored in the past few years through multiple strategies. The objective was not only to combine the physico-chemical properties of MOFs (porosity, crystallinity and functionality) with those of polymers (mechanical/chemical stability, processability and stimuli responsiveness) but also to impart novel features to the composite as a result of synergism between both components.^12,21,40^ A mutual recognition between MOFs and polymers as a result of attractive interactions at the MOF/polymer interface can influence both the structure and physicochemical properties of MOFs and polymers. This part will cover the synthesis of functional composites through the integration of polymers either in the framework or the pores of MOFs (Fig. 2b and c). We will discuss how the combination of MOFs with polymers can endow them with enhanced functionalities, thus expanding their practical applications. While polymers can be used for the design of MOFs, the opposite has also been successfully performed. Indeed, the structural and physico-chemical properties of MOFs can influence the controlled synthesis and separation of polymers. Given the particular attention that this aspect has received, it will not be covered in our perspective article and we guide the reader to relevant reviews.^12,40,41^

**Fig. 4:**
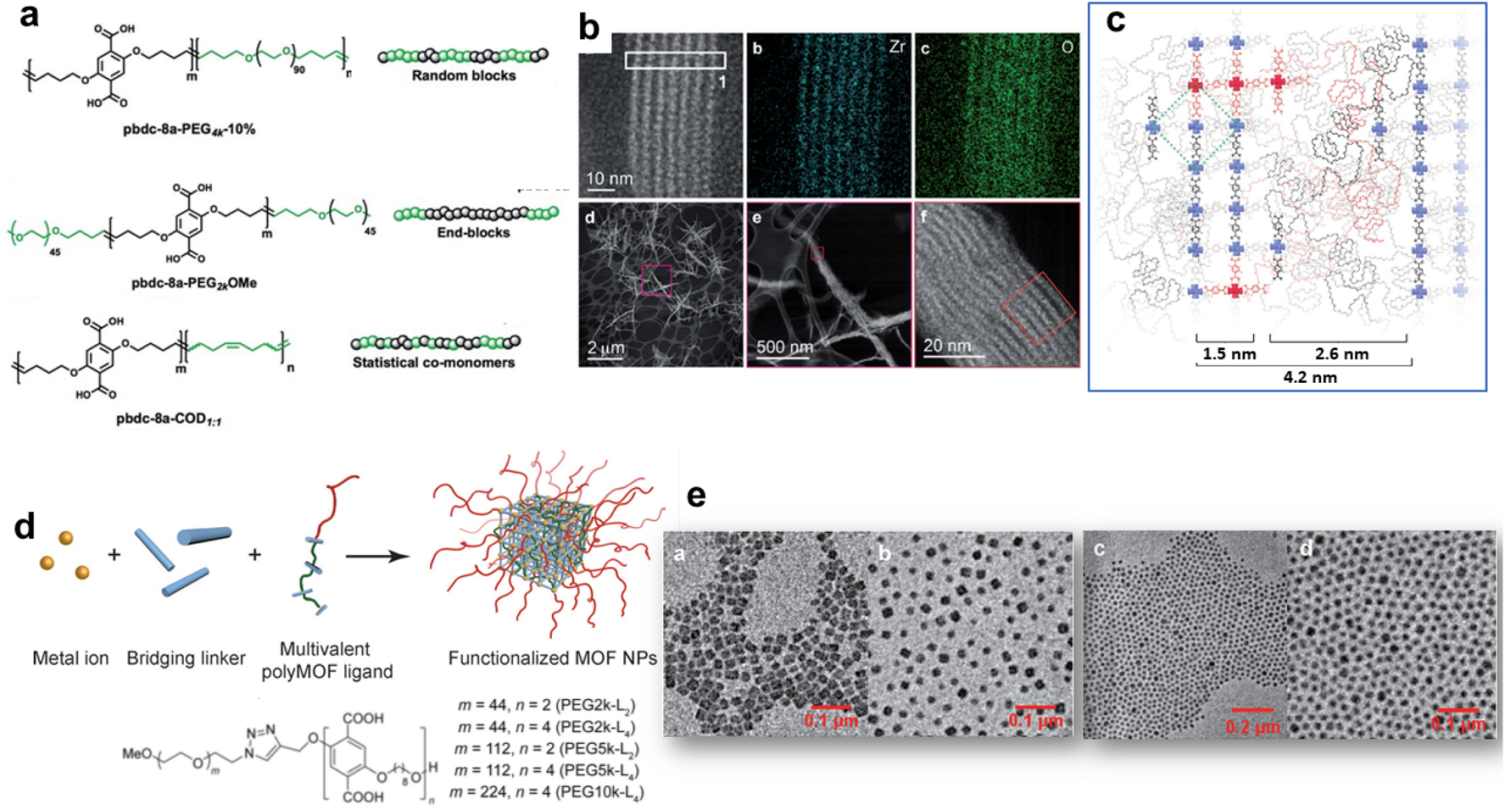
Design of polyMOFs. (a) Polymers used for the synthesis of polyUiO-66, (b) HAADF-STEM and elemental mapping of polyUiO-66 derived from pbdc-8a-PEG_4k_-10%, (c) reconstruction of layered assemblies in polyUiO-66 derived from pbdc-8a-PEG_4k_-10% (adapted with permission from ref. 46 Copyright 2020, Royal Society of Chemistry). (d) Strategy to develop poly-MOF-5 NPs, and (e) TEM images of poly-MOF-5 NPs formed with different block copolymer ligands (adapted with permission from ref. 48 Copyright 2019, Wiley-VCH).

The design of MOFs by using polymers as organic ligands was initially pioneered by Cohen and coworkers in 2015 (Fig. 2b).^42^ The strategy of converting a non-porous, flexible and amorphous polymer into a porous and three-dimensional crystalline network is entropically challenging since the packing and entanglements of polymer chains generally lead to polymer materials with low crystallinity and porosity. Moreover, the formation of MOFs with high crystallinity is generally favoured by using small and rigid organic linkers. Interestingly, this class of novel porous hybrid materials termed polyMOFs was able to inherit the complementary features of both polymers (good film-forming properties and processability) and MOFs (crystallinity and permanent porosity).^21,42^ To achieve this objective, a series of pbdc-*x*a polyethers containing benzene dicarboxylic acid (bdc) as repeating units connected by different methylene spacers (*x* = 5–8 carbon atoms) were synthesised by step-growth polymerisation and their hydrothermal reaction with Zn^2+^ salts produced a series of polyMOFs (*i.e.* Zn-pbdc-*x*a) isostructural to MOF-5.^42^ The bdc units served as organic ligands of the Zn-pbdc-*x*a. The crystallinity of such materials strongly depends on the length of the methylene spacer, the crystallinity being higher for *x* = 7–8.^42^ In addition, the hydrophilic–hydrophobic balance of such materials could be tuned by varying the spacer length. As a consequence, polyMOFs prepared with *x* = 7–8 were significantly more hydrophobic and chemically stable towards moisture than the parent MOF-5.^42^ While the pure pbdc-*x*a polymer ligands are non-porous, Zn-pbdc-8a and -7a exhibited a high residual BET area (856 and 1104 m^2^ g^−1^ for *x* = 7 and 8 respectively *vs.* 2960 m^2^ g^−1^ for the pristine MOF-5).^42^ The CO_2_ adsorption capacity of Zn-pbdc-7a and -8a is significantly higher than that of the pure MOF-5 and this may be explained by strong interactions between CO_2_ and the polyMOFs as a result of the confinement effect of CO_2_ molecules in the reduced porosity of such polyMOFs.^42^ In an attempt to extend the design of polyMOFs to other MOF architectures, new types of polyMOFs were developed by combining the pbdc-*x*a polyethers with co-ligands bpy (bpy = 4,4′-bipyridine) and bpe (bpe = 1,2-bis(4-pyridyl)ethane).^43^ Note that these bpy and bpe ligands were previously used for the construction of pillared square grid MOFs such as [Zn_2_(BME-bdc)_2_(bpy)]_*n*_ (MOF 1). This co-assembly of pbdc-*x*a with different “pillaring” pyridine co-ligands (pby and bpe) and metal cations (Zn^2+^ and Cu^2+^) successfully led to the formation of polyMOFs isostructural to pillared square grid based MOFs.^43^ This study clearly showed the potentiality of the polymer ligand approach to enlarge the family of polyMOFs. This was further demonstrated through the design of polyMOFs based on the chemically robust UiO-66(Zr) MOF.^44^ While pure UiO-66 crystals generally present an octahedral shape, the polyUiO-66 materials were shown to present a different interlaced morphology and a hierarchical porosity that were certainly imparted by the well-defined pbdc-*x*a ligands.^44^ Indeed, pbdc-*x*a were prepared by acyclic diene methathesis polymerisation (ADMET) and were thus characterised by a higher molecular weight and an improved dispersity in comparison to previous polymer ligands.^44^ Interestingly, it was shown that polyUiO-66 materials were not only obtained from homopolymers but were also formed from different block copolymers (random blocks and end-blocks) and statistical copolymers comprising coordinating pbdc blocks and non-coordinating poly(ethylene) (PEG) blocks (Fig. 4a). The crystal morphology of polyUiO-66 was shown to vary depending on the composition and architecture of the block copolymer ligands.^45^ However, the characterisation of the internal structure of this series of polyUiO-66 by coupling ultramicrotomed TEM, HAADF-STEM, STEM-XEDS and SAXS revealed that all polyUiO-66 presented a layered structure consisting of alternating nanolayers of metal-rich crystalline domains and metal-deficient non-crystalline polymer domains (Fig. 4b and c).^46^ This phase separation is likely due to the exclusion of non-coordinating PEG from the polyMOF pores.^46^ However, it can be entropically assumed that the individual polymer chains extend throughout the material and that multiple polymer chains contribute to the coordination at each Zr-oxo cluster. The preparation of polyMOFs from block copolymers was also reported by J. A. Johnson and coworkers.^47,48^ In particular, an innovative strategy was deployed to simultaneously achieve the size control of MOF nanoparticles and their surface functionalisation in one single step (Fig. 4d).^48^ In MOF chemistry, the control over the crystal growth of MOF usually proceeds through coordination modulation and thus involves the co-addition of a modulator such as a monodentate ligand with the precursors of the MOFs.^49^ The modulator can thus impact the kinetics of nucleation and growth process of MOFs by competing with the organic linkers for metal coordination. The strategy proposed by J. A. Johnson *et al.* consisted of using diblock copolymers where one short block contained multiple bdc sites for coordination modulation and a second long block was a linear PEG chain for surface functionalisation (Fig. 4d).^48^ It was shown that polyMOF-5 NPs with particle diameters ranging between tens to hundreds of nanometres could be isolated, the size of particles being smaller by increasing the length of the PEG chain (Fig. 4e).^48^ Note that the controlled formation of polyMOF-5 NPs presumably involved a self-assembly process between Zn^2+^, terephtalate and polyMOF ligands into micellar aggregates where a loosely coordinated core was protected by a PEG corona. Notably, the presence of the PEG corona significantly enhanced the long-term water stability and colloidal stability of polyMOF-5 NPs.^48^ Most of the previously reported polyMOFs were obtained by using polymer ligands prepared by step-growth polymerisations, multistep iterative exponential growth or under non-living cationic polymerisation conditions. J. A. Johnson explored the advantages of using polymer ligands prepared by radical polymerisation (*i.e.* reversible addition fragmentation chain transfer (RAFT)).^50^ In comparison to other polymerisation routes, RAFT offers the advantages of a higher control over the composition, molecular weight and dispersity of polymers.^50^ The use of well-defined RAFT-based polymers not only allowed the design of polyMOF-5 with tunable microstructural and textural properties but also showed that increasing the polymer dispersity favoured the polyMOF formation.^50^ Therefore, the synthesis of polyMOFs by using RAFT-based polymers with low-dispersity required the co-addition of H_2_bdc for the crystallization of polyMOFs.^50^

**Fig. 5:**
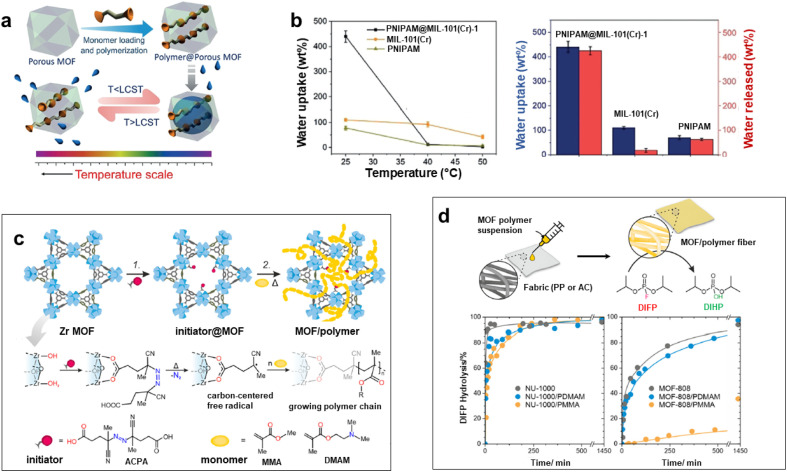
Design of MOF–polymer composites through the polymerization of monomers in the MOF pores. (a) Preparation of a composite combining MIL-101(Cr) and the thermo-responsive poly(*N*-isopropylacrylamide) (PNIPAM) and the temperature-triggered water capture and release process, (b) water uptake and water release of PNIPAM@MIL-101(Cr) in comparison to MIL-101(Cr) and PNIPAM under various conditions (96% RH for 25 °C, 40% RH for 40 and 50 °C) (adapted with permission from ref. 57 Copyright 2020, Wiley-VCH), (c) two-step protocol for the synthesis of Zr MOF (NU-1000 and MOF-808)/polymer hybrids. Step 1: solvent-assisted incorporation of a radical initiator. Step 2: free-radical polymerization in a MOF of acrylate monomers, and (d) preparation of Zr MOF/polymer/fibre composites and their catalytic activity for the hydrolysis of the nerve agent stimulant (DIFP) (adapted with permission from ref. 58 Copyright 2023, Royal Society of Chemistry).

Numerous studies reported the construction of functional composites through the integration of polymers in the pores of MOFs. A wide diversity of strategies was explored such as the infiltration of polymers in the MOF pores or the polymerisation of monomers from initiator-functionalized ligands within MOFs (Fig. 2c).^12,21,40^ The spontaneous polymer insertion into MOFs has been reported from the melting state or the solution state.^40^ Different polymers in the melt state such as PEG, linear alkanes or poly(methylpropylsilane) were included in the nanochannels of MOFs.^51–53^ This insertion proceeds presumably through the extension of polymer chains from coil conformation. It is thermodynamically favoured since the entropic loss upon polymer uncoiling is compensated by enthalpy gain as a result of attractive electrostatic and van der Waals interactions between the MOF network and the polymers.^40^ Polymer insertion into MOF pores also takes place under diluted conditions as a result of a competitive adsorption process between solvents and polymers in MOFs. This solvent-mediated polymer insertion into MOFs was equally reported for biomacromolecules such as proteins, DNA and single-stranded RNA. The main objective of protein inclusion into MOFs is to stabilize the enzymes and promote their catalytic activity.^54,55^ It is worth noting that this approach requires a size matching between the polymer chains and MOF pores since only polymers having a gyration diameter much lower than the MOF cavities are likely to enter the pores. Therefore, another strategy involving the post-synthetic polymerisation of monomers in the MOF pores was also extensively deployed (Fig. 2c). This generally implied a covalent grafting of polymers into the MOF backbone that was achieved through the insertion of an adequate functional group at the IBU or the organic linker of the MOF. Note that this approach can only be applied to chemically robust MOFs whose structure is not altered upon polymerization.^12,21^ As an example, B. Chen *et al.* reported the design of lanthanide MOFs (Ln-MOFs)/polymer membranes for the development of ratiometric luminescence thermometers.^56^ Ln-MOFs suffer from different shortcomings such as poor processability and very low chemical stability due to the lability of the Ln–O coordination bonds in aqueous solutions and acid/alkaline media. A mixed-lanthanide MOF (*i.e.* Eu_*x*_Tb_1−*x*_BABDC *x* = 0.0025) with the BABDC organic linker (BABDC = 2,5-bis(allyloxy)terephthalic acid) bearing polymerizable sites was thus synthesised and then copolymerised with butyl methacrylate monomers under UV and in the presence of a photo-initiator.^56^ This photopolymerisation process that occurs at RT under solvent-free conditions, allowed the formation of free-standing membranes with good mechanical properties. Remarkably, this MOF-polymer membrane retained the photoluminescence properties of the pristine Eu_*x*_Tb_1−*x*_BABDC MOF and behaved as an excellent luminescent thermometer capable of sensing temperature in the 90–240 K range.^56^ In contrast to the strong degradation of the pristine MOF upon water exposure, this membrane combined chemical stability and its performance in temperature sensing was not altered under harsh conditions such as high humidity and acid/alkaline media.^56^ D. Zhao *et al.* took advantage of the temperature-dependent coil-to globular conformational change of poly(*N*-isopropylacrylamide) (PNIPAM) to obtain thermo-responsive MOF–polymer adsorbents towards water (Fig. 5a and b).^57^ Upon conformational change, this thermo-responsive polymer undergoes a transition from the hydrophilic to the hydrophobic state. For that purpose, a series of composites were prepared by combining the mesoporous MIL-101(Cr) and PNIPAM with different PNIPAM loadings.^57^ First, the synthesis and activation of MIL-101(Cr) were followed by the encapsulation of NIPAM in the pores. The free-radical polymerization of NIPAM was carried out using azobisisobutyronitrile (AIBN) as the initiator.^57^ The full characterisation of these composites by multiple techniques was consistent with the accommodation of the polymer chains in the mesopores of MIL-101(Cr) while molecular modelling could reveal that the confined polymer chains are preferentially located in the large mesopores and mostly interact with the MOF backbone through hydrogen bonds.^57^ The water adsorption properties of such MIL-101(Cr)/PNIPAM composites were characterised by recording sorption isotherms at 25 °C and 90% relative humidity (RH). Notably, the water uptake of the MIL-101(Cr)/PNIPAM-1 composite with the lowest PNIPAM content was higher than that of the other composites due to a partial clogging of the MOF pores by the polymer chains upon increasing the PNIPAM loading.^57^ The adsorption performance of MIL-101(Cr)/PNIPAM-1 was finally tested in a humidity chamber at 96% RH and 25 °C. Remarkably, the MIL-101(Cr)/PNIPAM-1 composite was able to capture approximately 440 wt% of water under those conditions while the parent MIL-101(Cr) and pure PNIPAM only exhibited 110 and 74 wt% water uptakes respectively (Fig. 5b).^57^ The low water capacity of the pure PNIPAM and MIL-101(Cr) can be respectively attributed to the non-porous character of the polymer and the hydrophobic nature of the MOF. Finally, the phase transition of PNIPAM in MIL-101(Cr)/PNIPAM-1 was shown to trigger the release of about 98% of the captured water under mild conditions (40% RH and 40 °C) (Fig. 5b).^57^ In addition molecular dynamics simulations were performed to gain microscopic insight into the location and dynamics of water molecules in the composite as well as their interactions with both the MOF and polymers.^57^ This work sheds light on the design of stimuli-responsive adsorbents operating under mild conditions for atmospheric water harvesting application. In another example reported by W. Bury *et al.*, free-radical polymerisation was also used to design MOF/polymer catalysts for the degradation of chemical warfare nerve agents (CWAs) (Fig. 5c and d).^58^ These composites could thus be employed as active protective clothing and filters capable of detoxifying CWAs. Chemically robust mesoporous Zr MOFs (MOF-808 and NU-1000) were selected due to their high efficiency as catalysts for the hydrolytic degradation of CWAs. In the first step, a polymerisation initiator (4,4′-azobis(cyanovaleric acid), ACPA) was post-synthetically attached to the Zr-oxo clusters and in the second step, the free-radical polymerisation of vinyl monomers was carried out using these ACPA modified MOFs (Fig. 5c).^58^ The in-depth characterisation of such composites is consistent with the uniform distribution of the polymer chains in the MOF cavities. As a result of their good processability, these MOF/polymer hybrids could be easily deposited on polypropylene and activated carbon fabrics by drop-casting and the resulting MOF/polymer/textile composites presented a high catalytic activity for the degradation of a model nerve agent stimulant (Fig. 5d).^58^ Other methodologies were previously reported to introduce polymers in the pores of MOFs. As an example, a double solvent approach was used to preconcentrate dopamine in the pores of a Ni MOF, thereby inducing the selective dopamine polymerisation in the MOF pores while inhibiting this polymerisation reaction at the surface of MOF particles.^59^ Recently, W. L. Queen *et al.* took advantage of the CO_2_ adsorption capacity and the presence of accessible Cu(ii) catalytic sites of a Cu MOF (*i.e.* Cu-TDPAT with TDPAT^6−^ = 2,4,6-tris-3,5-dicarboxylphenylamino)-1,3,5-triazine) to prepare a novel Cu-TDPAT/polyazodianiline(PAD) catalyst for biomass conversion.^60^ After the loading of 4,4′-azodianiline monomers in the MOF pores, their subsequent reaction with CO_2_ molecules was catalysed by Cu^2+^ centres of the MOF and this led to the formation of short oligomeric chains confined in the pores of the MOF.^60^ Such oligomers consisted of azodianiline moieties bridged by urea linkages. Such composites were used as a catalytic support for the immobilisation and stabilisation of Pd NPs.^60^ This ternary Cu-TDPAT/PAD/Pd catalyst showed a high catalytic activity for the reductive amination of levulinic acid with a high recyclability.^60^ Such performance was partially explained by the dual stabilising effect of the polymer on both the MOF and Pd NPs. In numerous polymer-inside composites such as those presented in this part, the polymers were demonstrated to enhance the physico-chemical properties of MOFs (chemical, thermal or mechanical stability) or impart novel functions (processability, stimuli-responsivity, catalytic activity, conductivity, *etc*.). We would like also to stress that polymers can also alter the phase diagram of MOFs. This was previously reported for flexible MOFs. This class of MOFs is characterised by structural transformations between different metastable phases that can be triggered by different stimuli (uptake of guest molecules, temperature, pressure, *etc*.).^61^ This dynamic response of MOFs not only offers advantages in the selective adsorption of analytes for separation applications but also paves the way for the development of efficient sensing and delivery devices. The work reported by T. Uemura *et al.* illustrates the possible impact of polymer guests on the flexibility behaviour of MOFs.^62^ The flexible [Co_2_(2,6-ndc)_2_(bpy)]_*n*_ can exist in two forms: the activated MOF is a non-porous material that can be converted to a porous analogue upon solvent uptake. It has been shown that the incorporation of polystyrene in the MOF pores has stabilized the open phase of the MOF.^62^ The introduction of polystyrene into a Zn pillar-layered MOF has also been shown to alter the phase transformation of the MOF upon thermal treatment.^63^ Under those conditions the pure MOF evolved to a dense phase as a result of the pillar removal. In contrast, the thermal annealing of the MOF/polystyrene composites led to the formation of a turbostratic phase in which the Zn square-grid layers are threaded by polystyrene chains.^63^ These examples show that the assembly of MOFs with polymers can either increase the thermodynamic stability of MOFs or induce the formation of novel phases. A synergism between MOFs and polymers was thus observed in numerous polymer-inside composites presented in this section. The polymers not only impart mechanical properties and processability but could also enhance the chemical stability of MOFs and alter their dynamic and transport properties. Another class of MOF-based composites was developed relying on the integration of preformed MOF particles in different matrices as developed in the next two sections.

## Engineering of MOF surface functionality for the processing of composites

**Fig. 6:**
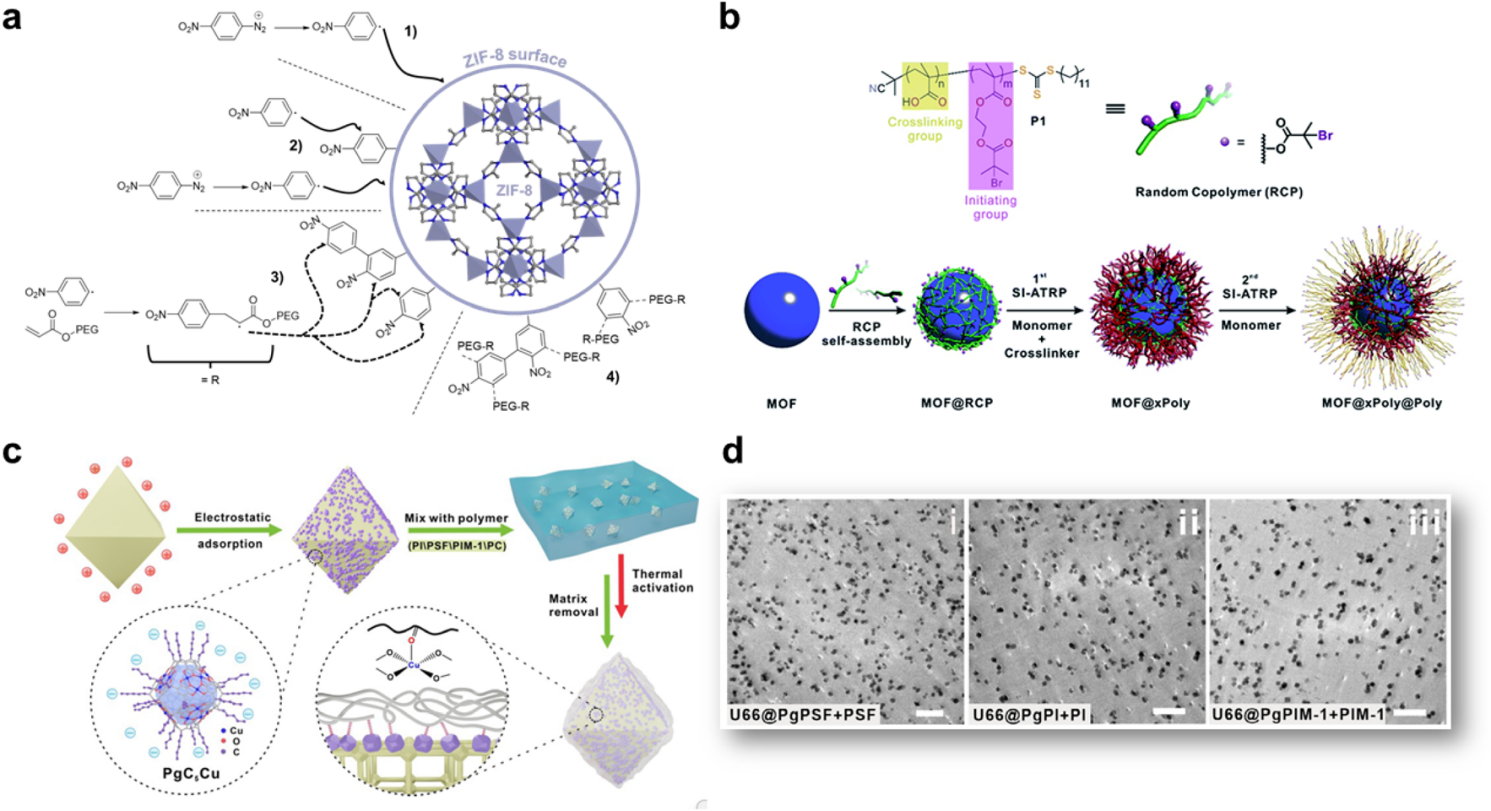
Strategies for the functionalisation of the outer surface of MOF particles. (a) Simplified scheme representing the supposed grafting mechanism of PEG onto the ZIF-8 external surface by Graftfast (adapted with permission from ref. 83 Copyright 2019, American Chemical Society), (b) schematic illustration of the growth of a polymer shell at the surface of MOF particles through SI-ATRP (adapted with permission from ref. 90 Copyright 2019, Royal Society of Chemistry), (c) schematic representation of the surface modification of MOFs by using a metal–organic nanocapsule, and (d) TEM images of ultramicrotomed slices of PSF, PI and PIM-1 modified UiO-66-NH_2_ dispersed in PSF, PI and PIM-1 respectively (scale bar = 1 μm) (c and d, adapted with permission from ref. 92 Copyright 2021, Wiley-VCH).

The insertion or *in situ* polymerisation of macromolecules in the pores of MOFs, as described in the precedent section is a reliable approach that led to the formation of functional composites. However, the inherent decrease in the surface area of MOF can have a detrimental effect on the transport or catalytic properties of MOF-based composites. Therefore, numerous composites based on MOFs were prepared through the blending of preformed MOF particles in different matrices ((bio)polymers, graphene, graphene oxides, carbon nitrides, *etc*) (Fig. 2d and e). The most widely used protocol for such composites consists of mixing a colloidal MOF solution with a polymer solution. After casting this mixture and solvent evaporation, a freestanding hybrid film is formed.^21,64^ Numerous MOF-based composites were also prepared in one step through the *in situ* approach involving the concomitant formation of MOFs and their co-assembly with the host matrix (Fig. 2e). These two strategies were widely used for the processing of hybrid membranes (also denoted as mixed matrix membranes, MMMs) in energy and environmental related applications.^64–66^ They were also developed for different bio-applications such as the design of scaffolds for bone tissue engineering and wound healing.^67^ Membrane separation has emerged as a promising alternative to cryogenic distillation or amine-based wet scrubbing for CO_2_ capture, with potentially high efficiency, lower energy consumption, ease of scale-up and environmental friendliness.^65^ MMMs were developed to potentially combine the gas transport and separation properties of the filler particles with the good processability and mechanical properties of the polymers.^68,69^ MOFs were selected as fillers due to their outstanding gas and liquid separation properties.^64^ However, numerous MOF-based MMMs suffered from several limitations that were mainly related to low chemical compatibility between MOFs and polymers.^64–66^ This issue is even more critical at higher filler loadings that are usually required to optimise the separation performance. Therefore, for numerous MMMs, MOF NPs could not be incorporated in sufficient quantity to establish a percolative network and the transport properties of MMMs were mainly dictated by the most abundant polymer phase.^64–66^ In addition, the aggregation or poor dispersion of MOF particles in the polymer matrix was prone to induce the formation of interphase defects that provided nonselective bypasses through the MMMs thereby compromising the mechanical properties, the selectivity and the separation efficiency of the membranes.^64–66^ While the design and applications of MMMs are outside the scope of this article, the reader can find a detailed coverage of MMMs in recent reviews.^64–66,70^ This section is intended to describe recent strategies deployed to enhance the interfacial properties between MOFs and polymers through a surface functionalisation of MOF particles. Engineering the surface functionalisation of MOFs can impact their physicochemical properties such as surface energy, colloidal and chemical stability, and adsorption properties. The coupling of polymers at the surface of MOF NPs with biocompatible polymers (cyclodextrin, chitosan, heparin, and poly(ethylene glycol) (PEG)) was envisaged in biomedicine to improve their colloidal stability in physiological media and the pharmacokinetic profile of drugs.^71–74^ However, in spite of the improvements achieved in these pioneering studies, this strategy often involved physical adsorption of polymers and thus precluded their strong attachment at the surface of MOF NPs, thereby leading to a low or non-controlled amount of polymer, a partial release of polymers in solution or a possible partial accommodation of polymer chains in the pores of MOFs. Therefore, a selective covalent grafting of a thin organic or polymeric layer at the outer surface of MOFs with a good control of the surface density of functional groups was considered as the most appealing surface modification of MOF NPs. Note that this surface modification of MOFs is challenging since it should be restricted to the external surface of MOFs while keeping intact the internal surface of MOFs and preserving their crystallinity and porosity. However, the difficulty relies also on the surface chemistry of MOFs which is inherently heterogeneous as a result of the presence of organic and inorganic building units, vacant coordination sites, surface defects and coordinating solvent molecules.^74^ Two covalent routes namely grafting-to and grafting-from, were extensively explored for the synthesis of polymer-grafted MOFs.^12,21^ The principle of the grafting-to approach consists of using presynthesised polymers with chain-end functional groups able to react with complementary reactive groups present at the MOF surface. This strategy thus took advantage of covalent interactions involving externally exposed functional sites of MOFs such as coordination unsaturated sites (CUS) or functional groups of the organic linker.^75^ Terminal phosphate-modified biomolecules such as cyclodextrins, nucleotides or lipids were covalently attached at the surface of MOF NPs through metal–phosphate coordination bonds, leading in some cases to a significant improvement of the colloidal stability of MOF NPs.^76–79^ Another strategy consists of introducing active unsaturated or nucleophilic functions in the organic linker that can be coupled to specific functions of polymers through carbodiimide-mediated amidation or click chemistry.^80–82^ As an example, R. S. Forgan and coworkers reported a selective two-step PEGylation approach of MOF NPs.^82^ UiO-66(Zr) NPs were first synthesised with azide functionalised modulators to control their particle size and their surface functionality. The covalent coupling of azide-functionalised UiO-66(Zr) NPs with propargyl-PEG chains was then performed by “click” chemistry through copper(i)-catalysed azide-alkyne cycloaddition.^82^ This PEG surface modification of UiO-66 NPs not only conferred an improved chemical and colloidal stability under physiological medium conditions but was also able to enhance the drug release kinetics and induced a pH-responsive release of the drug.^82^ Our group reported the synthesis of PEGylated ZIF-8 NPs through the Graftfast strategy.^83^ Graftfast^®^ also termed the diazonium-induced anchoring process is a versatile functionalisation approach that was developed to covalently graft a wide range of polymers with a defined thickness onto a large series of materials (silica, metal, plastic, ceramics, NPs, *etc*.).^84,85^ Moreover, this simple and single-step process occurs at RT and in water without any pre-modification of the MOF. It can thus be considered as a biocompatible, sustainable and scalable polymerization approach. Acryl-PEG chains of different lengths (480 Da and 5 kDa) were grafted onto the ZIF-8 external surface using GraftFast (Fig. 6a).^83^ This process was first induced by the chemical reduction of 4 nitrobenzene diazonium salt giving rise to surface-active 4-nitrobenzene radicals that could bind to the surface of ZIF-8 through carbon–carbon or/and carbon–metal bonds (Fig. 6a). The propagation of this reaction, with vinyl functional groups in solution, allowed the formation of a heterogeneous grafted polyphenylene film at the surface of ZIF-8 NPs (Fig. 6a).^83^ The functionalisation of ZIF-8 NPs by PEG brushes not only preserved the crystallinity and porosity of ZIF-8 but also significantly enhanced the long-term chemical and colloidal stability of ZIF-8 NPs in water.^83^ It was thus possible to prepare defect-free membranes combining PEG-ZIF-8 NPs and polyvinylalcohol (PVA) that have shown high performance in isopropanol dehydration by pervaporation.^83^ PEGylated MIL-100(Fe) NPs were also prepared by GraftFast and have shown high chemical and colloidal stability in biofluids while exhibiting a high adsorption capacity of drugs.^86^ In general, the grafting-to approach has the advantage of using polymers that were fully characterised before their reaction with MOFs but can lead to polymer-grafted MOF NPs with a low polymer density. Alternatively, the robust “grafting from” polymerization route was also developed to grow polymer chains from pre-tethered active sites at the MOF surface.^87,88^ Such a method involves the reactivity of an initiator-carrying linker from which the polymer is grown. This method has the advantage of producing MOFs grafted with highly dense polymer brushes but their characterization is a complicated process requiring the degradation of the modified particles and the subsequent isolation and characterisation of the polymers. Moreover, in numerous cases, this strategy cannot prevent the inevitable polymer insertion in the MOF pores as a result of the presence of the initiator located on the MOF framework. A third type of polymer grafting strategy referred to as “grafting through” was also used and consisted of functionalizing the MOF surface with a monomeric group that was then incorporated into a growing polymer chain. Recently, nylon 6,6 was covalently attached to UiO-66-NH_2_ under mild conditions.^89^ Although nylon 6,6 is one of the most widely used polymers, especially in the textile industry, its covalent coupling with MOFs under mild conditions was rarely reported. In this example, a post-synthetic modification of UiO-66-NH_2_ with adipolyl chloride followed by an interfacial polymerisation step with the hexamethylene diamine monomers led to the incorporation of the MOF into the growing polyamide fibers.^89^ Such composites presented a good catalytic activity for the degradation of a chemical warfare stimulant. Although this interfacial polymerization is a mild and scalable method, it is only limited to amine-based MOFs and cannot be applied to any MOF structure. In most of the previous studies, the grafting-from and grafting-through strategies were mainly applied to radical polymerizable polymers (methacrylate, acrylate, and styrenic) that are not suitable to address several MOF/polymer compatibility issues. Ideally, a good physicochemical matching between MOFs and a polymer matrix could be achieved by using polymer grafts that are chemically similar to the polymer matrix. However, in the field of gas separation, MMMs are mainly based on a limited number of condensation polymers such as polyimide (PI), polysulfone, polycarbonate and polymers of intrinsic microporosity (PIM) and the surface modification of MOFs with these polymers is still highly challenging so far. The group of T. Li reported different generalizable and non-covalent strategies to modify the surface of MOFs.^90–92^ In a first approach, a series of MOF–polymer composites was prepared through surface-initiated atom transfer radical polymerisation (SI-ATRP).^90^ However, in contrast to the traditional SI-ATRP process, the methodology followed by T. Li *et al.* did not imply the pre-anchoring of the ATRP initiator on the MOF surface but relies on the deposition of a macroinitiator at the surface of MOFs NPs (Fig. 6b).^90^ A random copolymer bearing multiple carboxylic acid groups and bromoisobutyrate (BiB) functional groups was thus coated at the surface of MOF NPs. The presence of the carboxylic acid group promoted the formation of inter-chain hydrogen bond cross-linking at the surface of MOFs (Fig. 6b). Subsequent polymerisation was initiated through the BiB groups with a monomer and a cross-linker, leading to the formation of a robust polystyrene shell at the surface of MOF NPs.^90^ Based on the reversible-deactivation characteristic of ATRP, a second polymer layer could also be grown from the surface of the first polymer layer through another SI-ATRP step, increasing the functionality of the composites (Fig. 6b).^90^ This strategy was also applied to a series of five MOFs (*i.e.* UiO-66(Zr), ZIF-8(Zn), ZIF-67(Co), MIL-96(Al), and MIL-101(Cr)), thereby demonstrating the generalisation of this approach. Moreover, through a judicious selection of the monomer, it was possible to finely tune the hydrophobic–hydrophilic balance of the MOF.^90^ Another approach reported by T. Li *et al.* relies on a coordinative crosslinking between MOFs modified by a metal–organic nanocapsule (MONC) and polymers (Fig. 6c).^92^ Indeed, this group has developed Cu-based MONC (*i.e.* PgC_5_Cu) that were shown to coordinatively crosslink a large series of polymers through their exposed Cu open metal sites.^93^ Compared to covalent grafting, this coordinative crosslinking approach is more generalizable since Cu sites can interact with any polar coordinative functions such as hydroxyl, carboxylate, sulfonyl, *etc.* For that purpose, the surface of UiO-66-NH_2_ (Zr) NPs was first decorated with PgC_5_Cu as a result of attractive electrostatic interactions.^92^ The mixture of the PgC_5_Cu modified MOF with a polyimide (6FDA-DAM) under moderate heating induced the cross-linking of the polymer chains at the surface of MOF leading to the formation of a 10 nm thick polymer coating.^92^ This method did not alter the porosity of UiO-66-NH_2_ as the large size of PgC_5_Cu prevented it from entering the MOF pores. Moreover, this strategy could be extended to other MOFs (MOF-801, ZIF-8 and ZIF-67) and also other condensation polymers commonly used in gas separation (polysulfone, PIM-1).^92^ As previously explained in this section, the integration of MOFs into condensation polymers is still facing compatibility issues and this strategy was revealed efficient for this class of polymers. The dispersion of polymer grafted MOFs into polymer matrices was finally assessed by TEM observation on ultramicrotomed samples (Fig. 6d).^92^ As expected, excellent compatibility was observed for composites combining polymer grafts and a polymer matrix with the same chemical composition (Fig. 6d).^92^ In another example, T. Li *et al.* used amine-functionalised methacrylate polymer chains physically entangled within the MIL-101(Cr) framework as the covalent anchoring points to graft PI brushes at the MOF surface.^91^ However, this method has the disadvantage of significantly reducing the MOF porosity which is not always desirable depending on the targeted application. In another approach reported by J. Rzayev *et al.*, the interfacial properties of MOFs with PI were improved through the post-synthetic ligand exchange between MOF crystals and a poly(amic acid) (PAA).^94^ Half of the repeating units of this polymer contained the bdc units that are commonly used as an organic linker in MOF structures. A PAA/MOF-5 composite was easily formed by mixing a suspension of MOF-5 NPs with a solution of PAA in DMF.^94^ The hybridisation process first involves the exchange between surface MOF ligands and the PAA chains, leading to the coating of PAA chains at the surface of MOF-5 NPs. The interaction of polymer chains with different MOF crystals was prone to induce a cross-linking of MOF crystals through a PAA matrix.^94^ This approach allowed the preparation of highly porous composites with a high MOF loading exceeding 80 wt%. The rate of the reaction was shown to strongly depend on the number of exchangeable ligand sites at the MOF surface and thus on the MOF particle diameter.^94^ Moreover, this process was successfully extended to ZIF-8(Zn) but unfortunately, the synthesis of composites failed for UiO-66, presumably as a result of the high stability of the Zr–ligand bond and the low exchange rate with PAA.^94^

To conclude this section, a high diversity of synthetic strategies was reported to functionalize the outer surface of MOF particles and optimize the compatibility and loading of MOFs with polymers and carbon-based materials. Table 1 gives a summary of these main approaches and describes the nature of the interactions involved between the MOF and carbon materials.

**Table 1:** Summary of the synthetic strategies used for the surface functionalization of MOFs

Strategy	Interactions between MOFs and the carbon material	Physico-chemical features of MOF required	References
Grafting to	Covalent	MOFs bearing CUS or functionalized organic linker	80–83
Grafting from	Covalent	Initiator-functionalized MOF	87 and 88
Grafting through	Covalent	Monomer-functionalized MOF	89
SI-ATRP with macro-initiator	Hydrogen bonds	—	90
Coordinative cross-linking	Electrostatic interactions and coordination bonds	MOF functionalized by a metal–organic nanocapsule	92
Post-synthetic ligand exchange with polymers	Coordination bonds	MOF with labile metal–ligand bonds	94

## Nanostructuration and hierarchy at the morphology and composition levels

As described in the precedent section, valuable strategies for MOF surface functionalisation were developed to enhance the interfacial properties of MOFs with different matrices such as polymers, graphene, graphene oxides, *etc.* For the vast majority of the composites reported so far, their microstructure can be described as a random distribution and orientation of MOF crystals in the host matrices. However, the spatial organisation and nanostructuration of MOF-based composites can strongly impact their physicochemical properties in adsorption, catalysis and detection. As an example in the domain of MMMs for gas separation, isotropic MOF fillers are often randomly oriented in the polymer matrix and this can present a detrimental effect on the MOF loading and the gas transport properties.^64^ Indeed, for numerous MOF crystals, the channels/pores are not parallel to the gas diffusion direction. The hierarchy of MOF-based composites at the scale of porosity, morphology and composition is thus crucial for their performance in applications. This section will be devoted to the approaches proposed to control the morphology, spatial distribution and organization of MOF NPs within the host matrices.

**Fig. 7:**
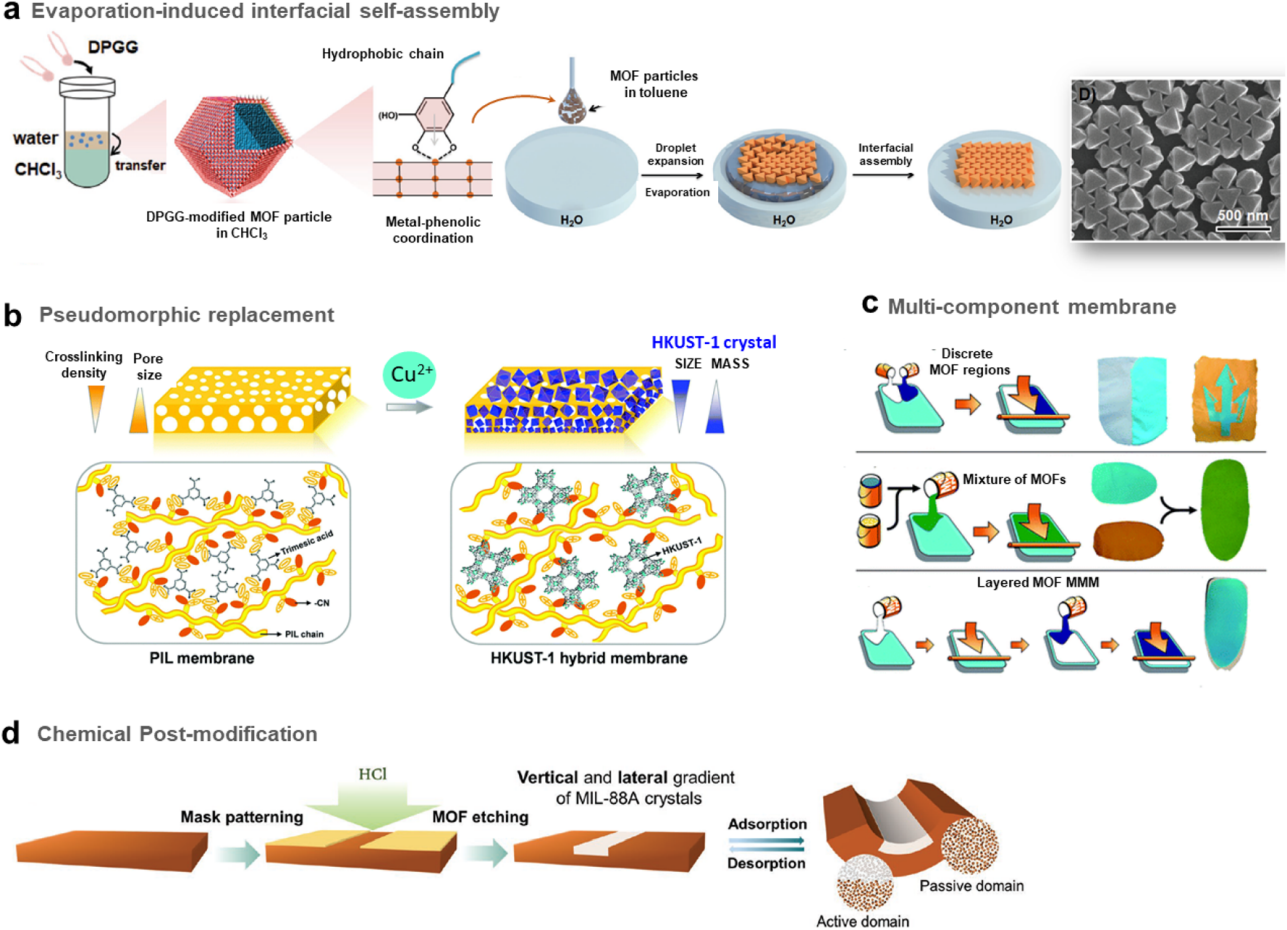
Different strategies to prepare MOF-based composites with a hierarchy in composition or spatial organization of MOF particles in the organic matrix. (a) Scheme of both the surface functionalisation of MOFs by the phenolic-based lipid molecule 1,2-dipalmitoyl-*sn*-glycero-3-galloyl (DPGG) (left) and the evaporation-induced self-assembly of DPGG-functionalised MOF particles on the air/water interface (middle). SEM image of DPGG-modified UiO-66 particles on the air/water interface (right) (adapted with permission from ref. 95 Copyright 2018, Wiley-VCH), (b) Schematic illustration of the pseudomorphic replacement approach for the preparation of an asymmetric HKUST-1 hybrid membrane (right) from a porous PIL membrane (left). The HKUST-1 hybrid membrane shows a gradient structure with increased mass and decreased crystal size from the top to the bottom part of the membrane (adapted with permission from ref. 97 Copyright 2017, Royal Society of Chemistry), and (c) strategies for the preparation of multicomponent MOF membranes: co-casting of different MOF inks (top), mixing of different MOF solutions (middle), successive casting processes of MOF inks leading to multi-layers of MOFs (adapted with permission from ref. 99 Copyright 2018, Royal Society of Chemistry). (d) Scheme of the patterning of MIL-88@PVDF films by chemical etching with HCl to produce asymmetric and self-folding membranes (adapted with permission from ref. 98 Copyright 2019, Wiley-VCH).

The group of C. J. Brinker reported a versatile functionalisation of the outer surface of MOFs through a phase transfer process involving the strong coordination of the surface metal centres of MOFs by the galloyl head groups of a lipid molecule (*i.e.* 1,2-dipalmitoyl-*sn*-glycero-3-galloyl, DPGG) (Fig. 7a left).^95^ This strategy was successfully applied to a large series of imidazolate or carboxylate-based MOFs (*i.e.* ZIF-8(Zn), ZIF-67(Co), UiO-66(Zr), MIL-88A(Fe), HKUST-1(Cu), MIL-101(Cr), CPP-3(In), and MIL-96(Al)). According to PXRD and N_2_ porosimetry, the crystallinity and porosity of MOFs are preserved after surface functionalization.^95^ As a result of the very high colloidal stability of the DPGG-modified MOF particles in the non-polar solvent toluene, MOF monolayers could be processed through evaporation-induced interfacial self-assembly (Fig. 7a right).^95^ Note that the assembly of MOF NPs into two-dimensional (2D) and 3D arrays can allow the integration of MOFs into well-defined periodic structures such as metamaterials, plasmonic or photonic-based sensors. As an example, it was possible to form a monolayer of ZIF-8 particles organized into a close-packed hexagonal array with a 〈111〉 crystal orientation.^95^ Compared to other MOF assemblies prepared by drop-casting, ZIF-8 NPs were more densely packed as a result of the clustering of hydrophobic MOF particles at the toluene/air interface. After toluene evaporation, the array was transferred to a water surface, allowing the processing of the freestanding film. Similarly, a hexagonal close-packing array of UiO-66 particles was obtained (Fig. 7a right).^95^ Freestanding ZIF-8 films supported on the poly(methylmethacrylate) (PMMA) support were also prepared by assembling DPGG-modified ZIF-8 with lipid-soluble PMMA added to the toluene/MOF solution.^95^ This assembly into 2D MOF arrays resulted from the phase separation of hydrophobic MOFs at the solvent/air and polymer/air interfaces.

It can also be of great interest to prepare MOF/carbon material composites in which MOF particles are heterogeneously distributed allowing the processing of asymmetric composites with a composition gradient. Such an approach has been deeply explored for the preparation of asymmetric MMMs for gas separation. These membranes typically consist of thin selective MOF–polymer layers supported on a nonselective porous polymer support.^65^ Such membrane configuration with a very thin selective layer (less than 1 μm) is required to optimize the membrane performance since the porous polymer layer can increase the gas flux and thus the gas permeability while the MOF–polymer layer can enhance the selectivity of the membrane.^65^ One of the main methods to manufacture asymmetric membranes is the phase inversion method. In this protocol, a MOF–polymer dispersion is initially cast onto a support and then immersed in a solvent inducing the precipitation of the polymer.^65^ As an example, Zhu *et al.* prepared asymmetric MMMs by assembling functionalised MIL-53(Al) particles and polyetherimide Ultem® 1000 polymer matrix.^96^ The post-functionalisation of MIL-53(Al) with aminosilane was required to enhance the compatibility between the MOF and the polymer.^96^

Hierarchy in the MMMs was not only created in their porous structure but also in the spatial distribution and size of MOF NPs. Indeed, the group of J. Yuan reported asymmetric HKUST-based MMMs exhibiting a gradient profile in their pore size, the diameter and distribution of MOF particles along the cross-section of the membranes.^97^ These membranes were prepared through a coordination-driven replacement procedure which consisted of using a preformed ionically cross-linked poly(ionic liquid) (PIL) membrane as a template and precursor for the processing of HKUST/PIL membranes (Fig. 7b).^97^ First, a membrane was fabricated through the deposition of a solution containing cationic PIL and trimesic acid (H_3_btc) (Fig. 7b left). The deprotonation of H_3_btc upon exposure of the membrane to NH_3_ solution induced the cross-linking of the membrane as a result of ionic interaction between btc^3−^ and PIL chains (Fig. 7b left).^97^ The gradual diffusion of NH_3_ in the membrane imparted a gradient both in its crosslinking density and porosity, with the pore size gradually increasing from the top to the bottom of the membrane. In the final step, this organic membrane was replicated by immersion into a Cu^2+^ solution, resulting in the formation of HKUST-1/PIL membranes that possessed size and mass gradient distribution of MOF crystals throughout the membrane (Fig. 7b right).^97^ It was finally shown that this membrane could exhibit a fast actuation in response to NH_3_ stimulus due to its intrinsic asymmetric microstructure.^97^ Post-treatment of MOF–polymer membranes was also performed to introduce a gradient of MOF NPs within the polymer matrix. D. Maspoch and coworkers reported patterned composite films that consisted of flexible MIL-88A(Fe) particles embedded in the PVDF (PVDF = poly(vinylidene fluoride)) host matrix (Fig. 7d).^98^ The spatial location of MOF crystals in lateral and vertical directions of the membrane was controlled through chemical etching with HCl vapours (Fig. 7d).^98^ Discrete regions of the membrane were specifically exposed to HCl vapours, leading to a complete degradation of the MIL-88A(Fe) crystals (Fig. 7d).^98^ The high flexibility of the MIL-88A(Fe) upon water adsorption was herein exploited to induce actuation in the etched MIL-88A(Fe)/PVDF films.^98^ Indeed, such patterned composite films exhibited a tunable self-folding response driven by water adsorption. Such responsive composite films could be used as autonomous soft mechanical devices in different applications such as micromanipulation and robotics.^98^

The introduction of different MOF particles within a single polymer matrix is also a valuable approach for the spatial compartmentalisation of different MOF catalysts within a single catalytic membrane. Such membranes may be used in flow system reactors for cascade reactions or as a multifunctional catalyst able to degrade a variety of different species in an industrial waste stream. In this context, the group of S. M. Cohen explored different strategies to prepare multi-MOF membranes with different microstructures and spatial distribution of MOF NPs (Fig. 7c).^99^ First, two MOF–polymer dispersions were deposited side-by-side at two different locations of the same substrate, leading to the formation of a continuous film after the solvent evaporation (Fig. 7c top). The high viscosity of the MOF–polymer suspensions inhibited the mixing of both colloidal suspensions on the substrate except at the interface between both solutions. This method thus led to a MMM with a total MOF content of 60 wt% constituted of two macroscopic discrete regions namely a UiO-66/PVDF domain separated by another HKUST/PVDF domain.^99^ In comparison, a mixed MOF MMM with a total MOF content of 60 wt% was prepared by casting a suspension containing the PVDF polymer with two MOFs (Fig. 7c middle). This bi-component MMM consisted of a random and homogeneous distribution of both MOF particles in the PVDF matrix without any phase separation and aggregation.^99^ This approach was applied to a large series of MOFs. Finally, MMMs with a multi-layered microstructure were prepared taking advantage of the slow evaporation of MOF/polymer suspensions due to the high boiling point of the DMF in such suspensions (Fig. 7c bottom).^99^ However, attempts failed to prepare such layered membranes by casting a second layer on top of a preformed layer and this was due to a partial redissolution of PVDF from the first layer. To overcome this issue, PVDF in the MMM was crosslinked by adding a small amount of a short linear diamine (*i.e.* hexamethylene diamine) to the MOF/PVDF suspension prior to casting of the MMM.^99^ To avoid any significant volume variation of the first layer, a pre-swelling of the PVDF within the first layer was required prior to the deposition of the second layer. This strategy led to the processing of two-layer and three-layer MOF MMMs with a vertical distribution of MOFs within the single PVDF matrix.^99^ Finally, the potential of mixed and layered MMMs as catalytic membrane reactors for chemical transformations was finally evidenced.^99^

**Fig. 8:**
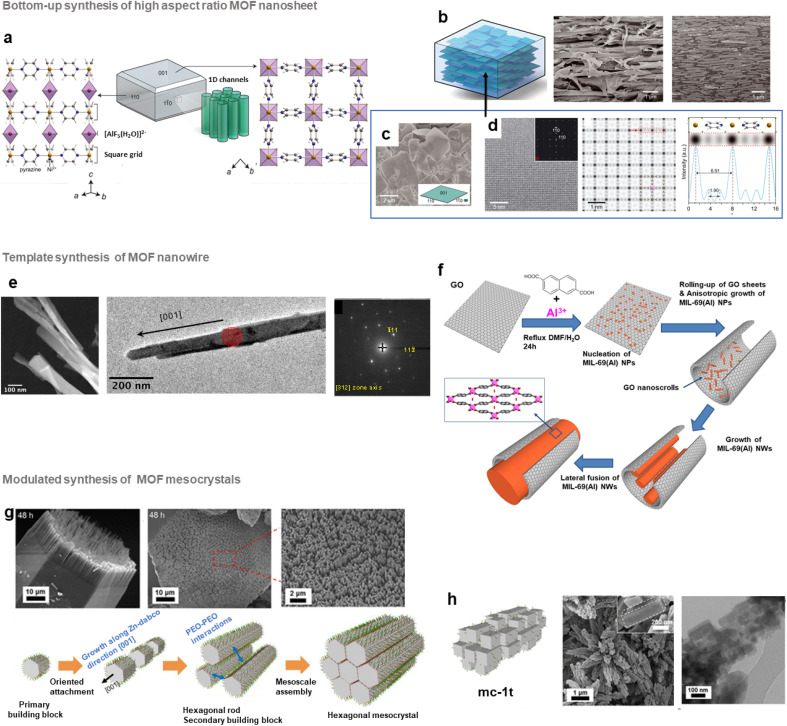
Different approaches reported to control the morphology of MOF particles in MOF-based composites. (a–d) Synthesis of AlFFIVE-1-Ni nanosheets and [001] oriented AlFFIVE-1-Ni/6FDA-DAM MMMs, (a) crystallographic structure of AlFFIVE-1-Ni along the [110] and [1–10] directions, (b) scheme (left), cross-section SEM image (middle) and focused ion-beam SEM image (right) of the [001] oriented AlFFIVE-1-Ni/6FDA-DAM MMM (c) SEM and (d) Cs-corrected STEM image of AlFFIVE-1-Ni nanosheets: ABF image along [001] with the Fourier diffractogram (left), the corresponding symmetry-averaged image (middle) and intensity profile along the red arrow (right) shown in the image (middle) (a–d, adapted with permission from ref. 104 Copyright 2022, Science), (e) HAADF-STEM (left), TEM-BF image (middle) and SAED (right) of MIL-69(Al)/GO NW, (f) summary of the main stages of the MIL-69(Al) NW formation (e and f, adapted with permission from ref. 111 Copyright 2020, Wiley-VCH), (g) SEM images (top) and the proposed growth mechanism (bottom) of mc-1h mesocrystals, and (h) schematic representation (left), SEM image (middle) and TEM image (right) of mc-1t mesocrystals (g and h adapted with permission from ref. 120 Copyright 2018, American Chemical Society).

In the past few years, different groups have developed highly oriented and layered composites by tuning the morphology of MOF nanocrystals. As an example, several MMMs composed of 2D MOF nanosheets with a pore structure parallel to the gas pathway were shown to exhibit excellent gas separation performance.^64,100^ This strategy thus relies on the design of thin MOF nanosheets prior to their assembly with polymers. Top-down exfoliation and bottom-up strategies were reported for the synthesis of MOF nanosheets.^64^ Top-down exfoliation involves mainly the delamination of bulk layer-structured MOFs through different methods including sonication-assisted exfoliation, and mechanical or chemical exfoliation. These methods are thus restricted to layered-structured MOFs and the resulting exfoliated nano-sheets present generally structural defects as a result of the strong external forces applied during the exfoliation process. Moreover, since the exfoliation step is quite complex, costly and time-consuming, the large scale production of MOF nano-sheets is impeded by this strategy. In contrast, the direct synthesis of MOF nano-sheets by bottom-up approaches is highly suitable for their large-scale production.^64^ Various bottom-up synthesis methods were reported including interfacial synthesis, sonication-assisted synthesis, three-layer synthesis or surfactant-assisted synthesis. One of the pioneering studies in this field was reported by J. Gascon and coworkers who developed Cu-bdc nanosheets through a three-layer synthesis.^101^ Cu-bdc nanosheets with a lateral dimension of 0.5–4 μm and a thickness of 5–25 nm were prepared by using three solvent layers containing a mixture of two miscible solvents of different ratios (DMF and acetonitrile).^101^ The nucleation and growth of Cu-bdc nanosheets took place at the interface between one solution containing the Cu source and another containing the H_2_bdc organic linker.^101^ Cu-bdc was selected as the parent bulk MOF exhibiting a layered structure. These Cu-bdc nano-sheets were then combined with the Matrimid polyimide for the processing of MMMs (*i.e.* ns-Cubdc@PI).^101^ As clearly shown by tomographic focused ion beam scanning electron microscopy (FIB-SEM) images, the Cu-bdc nano-sheets were homogeneously dispersed and remarkably, they were preferably oriented parallel to the basal plane of the membrane, thereby exposing their pore system in the direction of the gas flux.^101^ This configuration significantly enhances the spatial distribution of such nanoparticles in the polymer matrix in comparison to MMMs prepared by using isotropic Cu-bdc nanocrystals (*i.e.* nc-Cubdc@PI). As a consequence of this oriented microstructure, the CO_2_/CH_4_ separation selectivity of ns-Cubdc@PI MMMs is 30–80% higher than that of the pure polymer membrane and 60–140% higher than that of nc-Cubdc@PI MMMs.^101^ Similarly, MMMs based on 4 wt% Cu-bdc nano-sheets and highly permeable polyimides (6FDA-DAM and PIM-1) have shown a significant increase of the CO_2_/CH_4_ selectivity in comparison to the pure polymers but at the expense of a strong decrease in CO_2_ permeability.^102,103^ It was thus highly desirable to design other MOF nano-sheet fillers with inherently enhanced gas separation performance. Recently, the group of M. Eddaoudi developed a series of highly efficient MMMs based on (001)-oriented AlFFIVE-1-Ni nanosheets with a MOF loading up to 60 wt% for the CO_2_/H_2_S/CH_4_ separation (Fig. 8b).^104^ AlFFIVE-1-Ni exhibits a three-periodic framework constructed by assembling a square-grid layer based on Ni^2+^ cations and the pyrazine ligand connected by [AlF_5_(H_2_O)]^2−^ anions in the third direction (Fig. 8a).^105,106^ This structure consists of 1D ultrasmall channels running along the [001] direction allowing the diffusion of CO_2_ and H_2_S while excluding the entrance of CH_4_ as a result of the molecular sieving effect.^105,106^ Thus, this MOF exhibited excellent separation properties for H_2_S/CH_4_ and CO_2_/CH_4_ in addition to its high chemical stability.^105,106^ However, AlFFIVE-1-Ni produced by hydrothermal synthesis consisted of micrometer-scale crystals and was thus not suitable for the processing of MMMs.^104^ Interestingly, this three-periodic MOF could be synthesized as monodisperse and high-aspect ratio (001) nano-sheets with a lateral dimension of 0.5–4 μm and a thickness of 20–50 nm (Fig. 8c and d).^104^ This was achieved without adding any surfactant or modulator and by controlling the physicochemical parameters (low concentration of the [AlF_5_(H_2_O)]^2−^ unit, moderate temperature and choice of solvents) of the synthesis.^104^ MMMs based on AlFFIVE-1-Ni nano-sheets and 6FDA-DAM were then prepared by solvent casting under conditions of slow solvent evaporation, thereby inducing the in-plane alignment of nanosheets in the polymer matrix (Fig. 8b). This remarkable orientation of the nano-sheets in the 6FDA-DAM was clearly observed by recording cross-section SEM and FIB-SEM images (Fig. 8b).^104^ This stacking of the nano-sheets parallel to the basal plane of the membrane has the advantage of significantly improving the MOF/polymer compatibility, allowing the processing of MMMs with nanosheet loadings up to 60 wt%. Such MOF loading could not be achieved by using isotropic AlFFIVE-1-Ni nanoparticles.^104^ The [001]-oriented AlFFIVE-1-Ni/6FDA-DAM MMM demonstrated an excellent and long-term separation performance for both CO_2_/CH_4_ and H_2_S/CO_2_/CH_4_ separations that was even higher than that of numerous pure polymer membranes and MMMs previously reported in the literature.^104^ Very recently, the controllable growth of ultrathin Zr MOF nano-sheets (*i.e.* NUS-8) was successfully reported through a one-step modulated synthesis in solution.^107^ Such nano-sheets were used for the preparation of ultrathin (20 nm) and highly oriented NUS-8 membranes in a large area (>200 cm^2^) that demonstrated a high performance for liquid separation and desalination.^107^ Note that oriented and polycrystalline MOF membranes (without any polymer) were also extensively studied in the past few years but this topic is not in the scope of the present article and can be found in a relevant review.^64^

Our group has recently reported MMMs exhibiting a layered microstructure due to the preferential orientation of porous coordination polymer (PCP) platelets.^108^ This PCP (labelled MIL-178(Fe)) exhibited interesting properties for CO_2_ separation such as high hydrothermal stability, ultra-micropores and polar OH groups acting as CO_2_ adsorption sites.^108^ This material was synthesised as sub-micrometer-sized platelets through an easily-scalable and environmentally friendly protocol without using any toxic reactant.^108^ MIL-178(Fe)/Pebax®-3533 MMMs with a MIL-178(Fe) loading up to 25 wt% have shown an enhanced CO_2_/N_2_ separation performance in comparison to the pure Pebax®-3533. Such properties are likely due to the homogeneous dispersion and preferential orientation of MIL-178(Fe) fillers in the polymer matrix, the absence of any interfacial defects, the increase of the CO_2_ solubility and the lower crystallinity degree of the Pebax matrix in comparison to the pure polymer.^108^

Alternatively, the control of the MOF nanocrystal morphology was achieved by using organic templates that not only guided the crystallisation of the MOF NPs but also imparted processability and mechanical properties to the composites. Such composites were often prepared by the *in situ* synthetic approach in which the nucleation and growth of the MOF occurred in the presence of the organic template. Diverse organic templates such as (bio)polymers, graphene or graphene oxide (GO) were previously used to template MOF materials since they possess a large variety of coordination functions and can spontaneously self-assemble into different aggregates such as micelles, nanostructures or mesophases. GO is the oxidised form of graphene bearing different oxygen functional groups such as epoxy and hydroxy groups on the basal plane and carboxylic acid functions on the edges of the sheet. As an example, X. Zhang and coworkers reported highly oriented and ultrathin MOF membranes consisting of 2D nanosheets supported on a porous tubular substrate.^109^ A thin layer of GO was employed to control and guide the oriented growth of MOF nanosheets, due to attractive interactions at the MOF/GO interface.^109^ Graphene-derived materials were also shown to direct the growth of 1D MOF nanostructures such as nanowires (NWs) and nanotubes. The pioneering work reported by M. Jahan and coworkers demonstrated that the *in situ* formation of MOF-5(Zn) in the presence of benzoic acid functionalised graphene (BFG) led to composite nanowires.^110^ Each single NW consisted of stacked MOF-5 nanorods intercalated by BFG nanosheets regularly distributed along the NW as shown by TEM and micro-Raman spectroscopy.^110^ The BFG sheets did not only act as a structure-directing agent of MOF-5 favouring the growth of MOF-5 in a direction perpendicular to the BFG basal but they were also integrated as a component of the NW.^110^ Our group has also reported the synthesis of Al^3+^ dicarboxylate MOF (that is MIL-69(Al)) NWs by using GO nanoscrolls as a structure agent (Fig. 8e).^111^ We took advantage of the spontaneous scrolling of GO sheets in DMF into GO nanoscrolls as a result of the non-homogeneous distribution of oxygen basal functions of GO and surface tension.^111^ MIL-69(Al) NWs were synthesised *in situ* under reflux by mixing the precursors of MIL-69(Al) with GO as a co-reactant. This MIL-69(Al)/GO composite consisted of an assembly of GO nanoscrolls and MIL-69(Al) NWs with lengths up to 2 μm and an average diameter of 70 nm as shown by combining multiple techniques (PXRD, SEM, TEM, and HAADF-STEM) (Fig. 8e left and middle).^111^ According to selected-area electron diffraction (SAED) (Fig. 8e right), it was shown that each NW is a single crystal of MIL-69(Al) growing along the [001] direction which is parallel to the chain axis of Al^3+^ octahedra in the structure of MIL-69(Al).^111^ By coupling various *ex situ* characterisation tools and molecular modelling, it was shown that the anisotropic growth of these MOF NWs occurred in the inner cavity of the GO nanoscrolls and was certainly driven by covalent interactions between surface functional groups of MIL-69 (Al) and oxygen groups of GO (*i.e.* hydroxy or carboxylic acid functions).^111^ This material thus resulted from a mutual recognition between GO and the MOF: GO guided the 1D growth of the MOF while the MOF induced the structuring of GO. A mechanism for the formation of this composite was proposed involving mainly three steps: (i) the nucleation of MIL-69(Al) NPs and the concomitant rolling-up of GO sheets, (ii) the growth of primary small aligned MIL-69(Al) NWs in the inner cavity of GO nanoscrolls and (iii) the lateral fusion of MIL-69(Al) NWs into one single crystal MIL-69(Al) NW through the Ostwald ripening process and/or oriented attachment mechanism (Fig. 8f).^111^ We have also shown that this MIL-69(Al)/GO composite exhibited a semi-conducting behaviour although MIL-69(Al) is an insulating material. This indicated that the distribution of MOF NWs within GO did not disrupt the carrier traveling through the extended conjugated network in the GO sheets. This strategy of using GO nanoscrolls as MOF templates can thus be considered as a valuable process to homogeneously disperse MOF nanocrystals into GO matrices without any MOF aggregation while imparting good electron transport properties. Note that other carbon-based materials were used to template the formation of 1D nanostructures and superstructures based on MOFs and the reader can find a scope of this topic in a recent review.^112^ A large diversity of MOF/GO composites were prepared to circumvent a few drawbacks of MOFs including poor electron or thermal conductivity, low processability as well as a moderate chemical or thermal stability for some of them.^112^ Such composites were thus explored for different applications including supercapacitors, batteries, gas storage and catalysis. As an example, C. Serre and co-workers developed MOF/GO composites as adsorbents for CO_2_ capture.^113^ Although numerous MOFs present very interesting CO_2_ adsorption properties, they suffer from low thermal conductivity and thus slow desorption kinetics. This is critical for their application in the thermal swing adsorption process which requires a uniform and fast heating of the adsorbent during the desorption step.^113^ In this work, MOF/GO composites were thus explored for CO_2_ capture by microwave swing adsorption (MSA). Indeed, it was previously demonstrated that microwave irradiation can induce a faster CO_2_ release compared to conventional heating. MOF/GO composites were thus designed to combine the good CO_2_ adsorption properties of MOFs with the high electrical conductivity of GO. The Ti(iv) bisphosphonate MOF (*i.e.* MIL-91(Ti)) was selected due to its high CO_2_/N_2_ selectivity, good CO_2_ adsorption capacity and high hydrothermal stability.^114^ Two types of structurally different MIL-91(Ti)/GO composites with different MIL-91(Ti) loadings were prepared. First, MIL-91(Ti)/GO composites were prepared by a post-synthetic route involving the assembly of preformed MIL-91(Ti) NPs with GO sheets while a second series of composites were prepared using a one-step *in situ* approach.^113^ The objective was to compare the impact of the microstructure of such composites on their physicochemical properties. Only MIL-91(Ti)/GO *in situ* composites with a MOF loading as low as 5 wt% were very promising for CO_2_ capture by MSA.^113^ Indeed, such composites combined a high porosity, a high CO_2_ adsorption capacity and a semi-conducting behaviour while the MIL-91(Ti)/GO post-synthesis composites and the pristine MIL-91(Ti) were insulating. This indicated that both types of composites exhibited a distinct microstructure in which the MIL-91(Ti) NPs are spatially differently arranged on the GO sheets.^113^ According to the characterisation of the MIL-91(Ti)/GO *in situ* composite by combining XPS and TEM, it could be assumed that the nucleation and growth of MIL-91(Ti) NPs in such composites took place on the sp^3^ regions of GO sheets bearing coordinative oxygen functions that could interact with the Ti centres of the MOF.^113^ This nanostructuration of MIL-91(Ti) NPs on the GO sheets did not interfere with the electron hopping of GO taking place between the sp^2^ regions. This work highlights the importance of the strategy used to design a MOF-based composite.

**Fig. 9:**
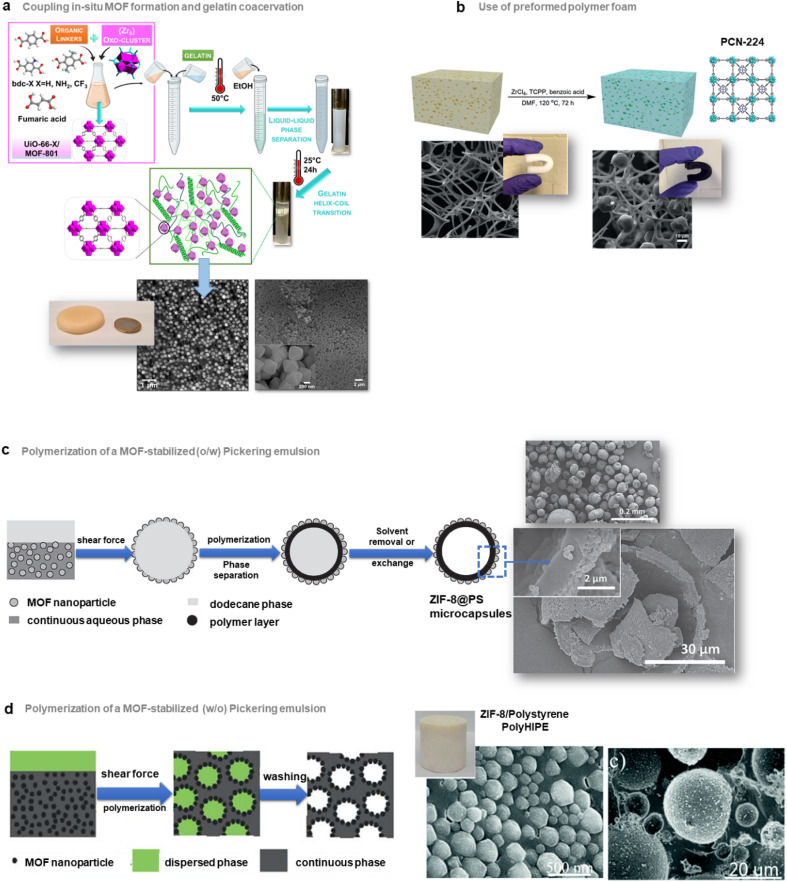
Strategies to impart hierarchical porosity in MOF–carbon material composites. (a) Approach coupling the *in situ* formation of MOFs with the gelatin liquid–liquid phase separation (coacervation) process (top), photograph of a shaped UiO-66/gelatin tablet (bottom left), TEM image of an ultramicrotomed slice of the UiO-66/gelatin hydrogel (bottom middle), SEM image of the washed UiO-66/gelatin hydrogel (bottom right) (adapted with permission from ref. 117 Copyright 2022, American Chemical Society), (b) procedure for the preparation of the PCN-224 decorated melamine foam composite (top) with the corresponding photograph and SEM image (bottom right) as well as those of the pristine melamine foam (bottom left) (adapted with permission from ref. 148 Copyright 2018, Wiley-VCH), (c) illustration of the MOF–polymer composite capsule formation (left) SEM image of ZIF-8@PS microcapsules (top right), SEM image (and inset) of a single broken capsule revealing the hollow interior (adapted with permission from ref. 153 Copyright 2013, Wiley-VCH), and (d) illustration of the interfacial assembly/polymerization procedure for the preparation of hierarchically porous MOF/polystyrene composites (left), photograph of a ZIF-8/PS monolith (middle) and SEM images of ZIF-8/PS composites (right) (adapted with permission from ref. 154 Copyright 2018, Royal Society of Chemistry).

Polymers and carbon-based materials were also shown to alter the kinetics of MOF crystallisation through coordination modulation. It is now well documented that the diameter of MOF crystals can be tuned by adding a molecular modulator during the synthesis of MOFs and different monodentate ligands such as monocarboxylic acids were frequently used.^49^ It is noteworthy that the dimension of MOF NPs is determined by a complex interplay between equilibria such as the organic linker deprotonation, the modulator deprotonation as well as the metal complexation.^115^ At high concentrations in the reaction medium, the addition of a molecular modulator can limit the deprotonation of the organic linker by decreasing the pH and its competition with the organic linker for metal coordination can allow a kinetic control of the MOF crystallisation, thereby leading to MOFs with large particle size.^49,115^ Alternatively, its introduction in the reaction medium can also reduce the size of MOF particles by acting as a surface capping agent.^49,115^ Numerous examples have shown the efficiency of monocarboxylic acids to control the diameter of UiO-type MOFs, and the addition of such molecules affects also the amount of structural defects, the porosity and eventually the hydrophobic–hydrophilic balance of such MOFs, thereby altering their adsorption or catalytic properties.^116^ Similarly, it was reported that polymers and biopolymers could exert the role of coordination modulators in the morphogenesis of MOFs as explained previously.^48^ Very recently, we have shown that gelatin can act as a modulator for the formation of UiO-66(Zr) NPs.^117^ Such MOF NPs were prepared by using an *in situ* approach in which the nucleation and growth of MOF NPs occurred in the presence of gelatin.^117^ Monodisperse and well-defined octahedral UiO-66(Zr) crystals with a diameter of 350 nm were synthesized in the presence of gelatin (Fig. 9a bottom) while UiO-66(Zr) NPs with an average diameter of 70 nm and a higher particle size distribution (40–200 nm) were obtained without gelatin and keeping constant the physicochemical parameters of the synthesis.^117^ This modulation effect was mainly attributed to the carboxylate side chains of the glutamate and aspartate residues of gelatin. Indeed, it was reported that amino acids such as l-proline could act as modulators for the formation of Zr and Hf MOFs.^118^ Moreover, gelatin was also shown to modulate the morphology of HKUST-1 crystals.^119^ B. V. K. J. Schmidt and coworkers reported that double hydrophilic block copolymers (DHBCs) could exert a remarkable role of crystal modulator and could induce the formation of MOF mesocrystals (Fig. 8g and h).^120^ In the past few years, these bio-inspired polymers were employed to control the crystallisation of different inorganic, inorganic/organic hybrid or organic crystals.^121^ They are made of one hydrophilic block increasing the water solubility of the polymer and another functional block interacting with a mineral surface.^2,121^ In this work, the DHBC selected (*i.e.* PEO_68_-*b*-PMAA_8_) consisted of one poly(methacrylic acid) block containing complexing carboxylic acid functions prone to coordinate metal ions and one solvating poly(ethylene oxide) block that can trigger mesoscale assembly.^120^ The effect of this polymer was studied with a 3D MOF, [Zn_2_(bdc)_2_dabco] (dabco = 1,4 diazabicyclo[2.2.2]octane)^122^ that can crystallise into a hexagonal metastable kinetic phase 1 h or a tetragonal thermodynamic phase 1t. Owing to its inherent metastable character, the control of both the dimension and morphology of 1 h has been rarely reported so far. Anisotropic hexagonal rod mesocrystals (mc-1h) with an average length and diameter of 2.9 and 0.53 μm were thus formed by adding PEO_68_-*b*-PMAA_8_ as a co-reactant into the reaction medium of bulk 1t (Fig. 8g).^120^ These rods consisted of unidirectionally aligned hybrid nanocrystals along their long axis as demonstrated by SEM and TEM analyses (Fig. 8g top).^120^ Electron diffraction of mc-1h shows the characteristic pattern of a single crystal, thereby indicating that the primary nanocrystals were perfectly aligned to a joint crystallographic system.^120^ Similar results were obtained with the Cu structural analogue MOF [Cu_2_(bdc)_2_dabco]. According to the time-dependent TEM and SEM experiments, the growth mechanism of mc-1h was proposed (Fig. 8g bottom). First, the fast oriented attachment of small NPs formed at the first stages of the reaction led to anisotropic secondary nanorods.^120^ PEO_68_-*b*-PMAA8 chains presumably adsorbed on the Zn-bdc surfaces could stabilize the nanorods of mc-1h. Then, the continuous growth of nanorods associated with their mesoscale assembly mediated by PEO blocks produced hexagonal mesocrystals (Fig. 8g bottom).^120^ In the absence of PEO_68_-*b*-PMAA_8_, the kinetic phase mc-1h evolved upon time to the thermodynamic mc-1t phase through redissolution-crystallisation processes. Therefore, the surface modification of mc-1h mesocrystals by PEO_68_-*b*-PMAA_8_ was able to hinder the transformation of mc-1h into mc-1t.^120^ The transformation of mc-1h into mc-1t was induced through the partial dissolution of PEO_68_-*b*-PMAA_8_ chains of mc-1h in methanol. This led to the crystallisation of mc-1t mesocrystals composed of hexagonally arranged cubic NPs (Fig. 8h).^120^ Remarkably, this MOF–polymer mesocrystal exhibited a high morphological complexity characterized by a dual hierarchical micro/mesoporosity and dimensionality. This work clearly outlined that DHBC could be very useful to control the crystallization of MOFs and also impart a hierarchical dimensionality and porosity as previously reported for biominerals, oxides, and organic crystals.^2,121^ This strategy could thus potentially expand the range of mesocrystals by using MOFs and DHBC of different structures and functionalities.

To conclude this section, a high diversity of MOF-based composites were prepared through the blending of MOF particles into a wide range of matrices ((bio)polymers, graphene, graphene oxide, carbon nanotubes, *etc*). Initially, the first generation of composites consisted of randomly distributed MOF particles within the matrices with a low to moderate amount. Through an adequate functionalization of MOF particles, MOF-based composites could be processed with a high MOF loading (>30 wt%). More recently, a second generation of hierarchically structured composites was developed through precise control over the loading, morphology and spatial positioning of MOF particles within the matrices. These materials were mainly explored in gas/liquid separation and catalysis. The next section will be focused on hierarchically porous MOF composites.

## Hierarchical porosity in shaped MOF-based composites

MOFs have shown great potential in applications related to gas/liquid separations, sensing or catalysis but these applications are generally limited to the adsorption, conversion or detection of small molecules as the majority of these materials are microporous. This range of pore size may decrease mass-transportation rates, limit the diffusion of substrates to catalytically active sites and finally prevent the processing of MOF composites embedding large components such as nanoparticles or proteins. To extend the porosity of MOFs in the mesoporous regime (pore size 2–50 nm), hierarchically porous (HP) MOFs have gained significant attention in the past few years.^123,124^ In addition to their intrinsic microporosity, these HP MOF frameworks show the peculiarity of containing an array of meso- and/or macropores in their framework. The construction of MOFs with intrinsic hierarchical porosity is outside the scope of this article and the reader can refer to recent reviews.^123–125^ In comparison to pure HP MOFs, the assembling of MOFs with (bio)polymers or carbon-based materials can introduce multiple inter-connected porosities over lengths in the micro-, meso-and macroporous regimes that can be located in the MOF framework, in the carbon-based matrix or at the MOF/carbon material interfaces. Different complex fluids (*i.e.* blends of polymers, (bio)polymer solutions, micellar solutions, colloidal suspensions, gels, foams and emulsions) were used not only to process MOF based composites in different shapes (monoliths, membranes, films, beads, *etc*.) but also to impart a hierarchical porosity (Fig. 2f).^126^ These complex fluids often present non-Newtonian fluid properties under an applied deformation (*e.g.* shear) including viscoelasticity, yield-stress, and shear-thinning viscosity that could be exploited for the processing of composites.

As an example, C. Janiak and coworkers developed MOF/PVA (PVA: polyvinyl alcohol) monoliths for applications in the field of adsorption heat pumps and thermally driven adsorption chillers.^127^ These composites were prepared through a non-solvent-induced phase separation. In this procedure, acetone as a non-solvent of PVA was added to an aqueous suspension containing PVA and preformed MOF particles (*i.e.* MIL-101(Cr) or Basolite A 520 (aluminium fumarate, Alfum)).^127^ Once the liquid–liquid phase separation was completed, it was possible to obtain a series of MIL-101(Cr)/PVA and Alfum/PVA composites with different MOF loadings.^127^ Composites with a MOF loading up to 80 wt% presented a high crystallinity and porosity according to PXRD and N_2_ porosimetry. For MIL-101(Cr)/PVA, the mesoporosity of the neat MOF was accessible to a large extent and almost 80% of the MIL-101(Cr) water uptake was retained.^127^ In contrast, Alfum/PVA composites lost partly the microporosity inherent in the MOF but exhibited mesopores presumably located at the MOF/PVA interface. Such mesopores led to an increase in the water adsorption capacity in comparison to the pure Alfum.^127^

A wide range of biomacromolecules including polysaccharides or proteins, have also been combined with MOFs to impart a dual micro-/mesoporosity.^128,129^ In addition to the intrinsic porosity of the MOFs, additional pores can be formed either at the biopolymer/MOF interfaces or in the biopolymer matrix. Biomacromolecules have also the advantages of presenting a high density of functional groups on their backbone and self-assembly properties for which no synthetic polymers are currently available. Moreover, their large abundance, non-fossil origin, good biocompatibility and biodegradability are key features for the development of “green” composites. Composite hydrogels were synthesised through the concomitant gelation of alginate and the *in situ* formation of MOFs.^130,131^ Alginate is a polysaccharide and a linear copolymer consisting of random sequences or blocks of 1,4-linked β-d-mannuronate (M) and α-l-guluronate (G block) residues. It is well known that a sol–gel transition of this biopolymer can be triggered by adding divalent or trivalent cations to an alginate aqueous solution. The exchange of the Na^+^ cations by divalent cations such as Ca^2+^ on the G blocks can induce a cross-linking of the alginate chains. MOF–alginate composites were thus prepared by a two-step procedure.^130^ First, alginate hydrogels were prepared by cross-linking the biopolymer with different divalent or trivalent cations (Cu^2+^, Co^2+^, Zn^2+^, and Fe^3+^) used as precursors of MOFs (*i.e.* HKUST-1(Cu), ZIF-67(Co), ZIF-8(Zn), and MIL-100(Fe) respectively). These hydrogels were post-synthetically treated with the organic linker of MOFs, leading to a series of MOF/alginate hydrogels.^130^ These composites could be processed as spherical beads by adding droplets of alginate aqueous solutions into the metal aqueous solutions. Although these composites presented a good MOF loading, they suffered from moderate porosity as a result of the penetration of alginate chains in the MOF pores.^130^ Moreover, ZIF-8 and ZIF-67-based composites had low chemical stability in water due to the low affinity of the Zn^2+^ and Co^2+^ cations for alginate.^130^ An alternative synthetic approach of the HKUST-1/alginate hydrogel was recently explored for which the btc^3−^ ligand was added to the Ca^2+^ exchanged alginate beads prior to the supply of Cu^2+^ cations.^131^ In comparison to the first strategy (*i.e.* addition of the btc^3−^ ligand to the Cu^2+^ exchanged alginate beads), the HKUST-1/alginate hydrogel presented a higher crystallinity due to the larger amount of HKUST-1 and an enhanced distribution of MOF particles in the alginate matrix.^131^ This composite has shown a good performance for the selective capture of dye. Among biopolymers, gelatin is one of the most widely used in industry due to its low cost and non-toxicity. Moreover, a wide range of nanocomposites were prepared by assembling gelatin with different inorganic materials (zeolites, clays, (hydr)oxides, and hydroxyapatite) and demonstrated a high efficiency in the field of regenerative medicine, biosensing or drug vectorization.^132,133^ The combination of MOFs with gelatin was recently explored for different bio-applications including tissue engineering, wound healing or drug delivery.^119,134,135^ The design of such MOF-gelatin hydrogels was mainly carried out through the blending of preformed MOF particles into either functionalized gelatin-based scaffolds or physical gelatin gels.^119,134,135^ Although such composites have shown promising properties, they present severe shortcomings such as low porosity and low MOF content which largely limits their practical application.^119,134,135^ Moreover, most MOF/gelatin hydrogels were synthesised by using MOFs with moderate stability while the processing of such composites with chemically robust MOFs is quite scarce. In this context, our group has reported a novel approach to design a series of chemically stable and highly porous MOFs–gelatin nanocomposites by taking advantage of the thermo-reversible behaviour of gelatin and a liquid–liquid phase separation, namely the coacervation process (Fig. 9a top).^117^ Coacervation is an efficient formulation process widely used in industry for the microencapsulation of drugs and cosmetics.^136,137^ Coacervation of gelatin is a long-established phenomenon that was explored from the thermodynamic and theoretical point of view by different groups.^138,139^ This is a liquid–liquid phase separation that takes place by adding either a non-solvent (simple coacervation) or polyelectrolytes (complex coacervation) to the gelatin solution. In our work, the simple coacervation of gelatin was induced by adding ethanol (nonsolvent) to an aqueous solution of gelatin (Fig. 9a top).^117^ Gelatin behaves like a polyampholyte in water due to the presence of negatively and positively charged amino acid residues. An aggregation of gelatin molecules is triggered by adding ethanol to an aqueous solution as a result of intermolecular charge neutralization of gelatin molecules.^140^ This leads to a phase separation between a gelatin-rich and a gelatin-poor solution phase (Fig. 9a top). One of the advantages of gelatin coacervates is their high gelatin concentration reaching values up to 45 wt% while it cannot exceed 15 wt% for physical gelatin gels prepared through the classical gelatin dissolution. In this study, hydrogels were formed by coupling the coacervation process of gelatin with the *in situ* formation of UiO-66-type MOFs (Fig. 9a).^117^ The RT synthesis of UiO-66(Zr)NPs previously reported^141^ was thus carried out by reacting the precursors of the MOFs (*i.e.* {Zr_6_} oxo cluster and the bdc^2−^ ligand) with gelatin aqueous solution and ethanol (Fig. 9a).^117^ After stirring this mixture for 24 h, a white viscoelastic hydrogel was obtained.^117^ Hybrid hydrogels were prepared in a similar manner by using a series of functionalised UiO-66-X(Zr) (X = NH_2_, 2CF_3_) as well as the Zr^4+^ fumarate MOF (*i.e.* MOF-801) (Fig. 9a).^117^ This indicates that this strategy can be applied to MOFs of different hydrophilic–hydrophobic balance and functionality while gelatin is a water soluble polymer. The characterisation of these hydrogel composites by coupling advanced characterization tools and molecular modelling has pointed out that they combine attractive features such as a high MOF loading (>80 wt%), homogeneous MOF distribution in the gelatin matrix and excellent MOF/gelatin interfacial properties (Fig. 9a bottom).^117^ As shown by N_2_ porosimetry, these composites are highly porous and present a bimodal micro- and mesoporosity. The microporosity of such composites inherent in the MOF framework is almost not impacted by gelatin, indicating that the micropores of the MOFs are not occupied by gelatin chains. These materials exhibited also mesopores that were presumably located at the interfaces between the MOF and gelatin or between MOF particles. These composites could be processed as coatings or thick tablets due to their good mechanical properties under humid ambient conditions (Fig. 9a bottom).^117^ Finally, these shaped composites have shown a high performance for the selective capture of acetic acid in the presence of humidity and could thus be used as adsorbents to tackle the air quality of indoor environments and the preservation of cultural heritage.^117^

To extend the porosity of MOF-based composites in the meso- and macroporous regimes, MOFs/(bio)polymers and MOF/GO gels were dried by either supercritical drying or freeze-drying.^124,126^ Such technologies were classically used for the processing of inorganic and organic macroporous aerogels.^142^ The principle of supercritical drying is to heat a wet gel at a temperature and pressure exceeding the critical temperature *T*_c_ and pressure *P*_c_ of the liquid entrapped (ethanol, CO_2_) in the gel porosity. The liquid is thus transformed to a supercritical fluid with a low surface tension and this drying allows preservation of the porous texture of the wet material quite intact by avoiding the pore collapse phenomenon.^142^ Alternatively, freeze-drying was also widely employed to prepare hierarchically porous MOF-based composites. In this technology, the solvent entrapped in the gel is first frozen and thereafter dried by sublimation. The frozen solvent acts as a porogen to generate large pores in the material.^142,143^ A wide range of aerogels were developed by different groups through the combination of MOFs with diverse (bio)polymers or carbon-based materials (graphene, graphene oxide, *etc*.).^124,126,144,145^ Their synthesis is generally based on the formation of a hybrid gel combining the MOF and carbon material followed by its supercritical drying or freeze drying. The hybrid gel can be prepared either by mixing preformed MOF particles or through the *in situ* synthesis of MOFs. In comparison to the direct mixing, the one-step *in situ* synthesis generally led to an enhanced distribution of MOF particles in the gel as previously explained. As an example, S. Kaskel and coworkers reported the synthesis of chitin–MOF composites with a hierarchical porous microstructure.^146^ Chitin is a chemically stable natural polysaccharide currently used for adsorption, filtration and water cleaning as well as bio-applications. Porous chitinous fibres extracted from a marine sponge were used as scaffolds to induce the *in situ* formation of the HKUST-1 MOF.^146^ This chitinous skeleton was thus exposed to a mixture of Cu^2+^ salt and the btc^3−^ ligand under physicochemical conditions similar to the RT synthesis of the pure HKUST-1.^146^ This step was followed by a supercritical drying with CO_2_ as supercritical fluid. According to PXRD and SEM, it was shown that HKUST-1 crystals were formed inside the hollow chitin fibres.^146^ HKUST-1/chitin presented a high MOF loading (53 wt%) and a remarkable BET surface area (800 m^2^ g^−1^ for the composite *vs.* 18 m^2^ g^−1^ for the pure chitin) due to the presence of the MOF micropores as well as an array of inter-particle mesopores and macropores.^146^ This hierarchical porosity confers to this material good gas transport properties and a high adsorption capacity of ammonia.^146^ The group of C. Li developed aerogels by assembling TEMPO-oxidised cellulose nanofibrils (TEMPO = 2,2,6,6-tetramethylpiperydil-1-oxyl) and divalent metal MOFs (ZIF-8 (Zn), ZIF-67(Co) and HKUST-1(Cu)).^147^ These materials combined the microporosity of MOF crystals with the bimodal meso- and macroporosities, processability and low density of cellulose aerogels.^147^ The advantage of using cellulose nanofibrils is related to their high surface area and the high density of carboxylate groups that can act as strong binding sites for MOF nucleation and growth. The preparation of such aerogels is based on the sequential processes of cellulose gelation, *in situ* formation of MOFs and freeze drying.^147^ As previously reported for alginate hydrogels, the sol–gel transition of cellulose proceeds similarly through cation-driven cross-linking of cellulose. The metal-chelated cellulose hydrogels were solvent-exchanged with methanol and finally exposed to the organic ligand, thereby leading to the nucleation and growth of MOFs.^147^ After freeze-drying, the MOF/cellulose aerogels exhibited a fibrous and multimodal porous structure.^147^ Due to their good mechanical properties, this composite could be shaped in different forms such as monoliths, tablets, *etc*… depending on the target application. They have shown a high adsorption kinetics and adsorption capacity for organic dyes.^147^ One of the main limitations of the preparation of aerogels through the *in situ* MOF synthesis is that it essentially applies to MOFs that can be obtained under mild ambient conditions. Indeed, such conditions are compatible with the chemical stability and gelation of the carbon based matrix. In general, solvothermal conditions used for the synthesis of MOFs are not applicable since they can lead to degradation and/or precipitation of the carbon based material. Moreover, this strategy is also restricted to chemically robust MOFs that are stable in the presence of the solvents (water and ethanol) used for the gelation of the (bio)polymer.

To circumvent such issues, the integration of MOFs into preformed 3D foams or aerogels was also envisaged to impart macroporosity in MOF based composites. The *in situ* formation of MOFs was carried out by mixing preformed aerogels of graphene, graphene oxide or biopolymers with the metal and ligand precursors of the MOF.^124,126^ One key point is to control the growth of MOF particles that can seriously impact the textural properties of the carbon aerogels. As an example, the group of H. -C. Zhou used preformed macroporous melamine foam (MF) as a scaffold to induce the one-pot *in situ* synthesis of a series of porphyrin PCN-224 MOFs (Fig. 9b).^148^ These highly stable MOFs are constructed by assembling {Zr_6_}oxo-clusters with metalloporphyrin based on the square planar tetrakis(4-carboxy phenyl)-porphyrin (TCPP) ligand (Fig. 9b right). Note that the TCPP ligand can be coordinated to various metal cations (M = Fe, Ni, Co, Ru), giving rise to a series of bi-metallic PCN-224 (M) MOFs. The series of PCN-224(M)/MF composites were prepared through the solvothermal treatment of a mixture containing the Zr^4+^ and TCPP(M) precursors as well as benzoic acid and MF (Fig. 9b).^148^ Remarkably, the macroporous scaffold of melamine was preserved under solvothermal conditions as shown by SEM and this network was homogeneously decorated with micrometre-sized PCN-224(M) crystals (Fig. 9b bottom).^148^ These PCN-224(M)/MF (M = Fe, Ni, Co, Ru) composites combined a high MOF loading, high crystallinity, hierarchical meso-/macroporosities, high chemical stability and good mechanical properties.^148^ They were finally tested for the catalytic epoxidation of cholesteryl esters in order to evaluate the impact of their hierarchical texture on their catalytic performance. PCN-224(Ru)/MF outperformed the other composites for this reaction as well as the pure PCN-224(Ru).^148^ PCN-224(Ru)/MF exhibited high catalytic activity for the epoxidation of cholesteryl esters with yields of more than 90% owing to the enhanced diffusion of substrates in its macroporous network. This composite could be recycled more than 6 times with good stability in its microstructural and catalytic properties.^148^

Emulsion templating is one of the most frequently employed strategies to prepare porous polymers and composites.^142^ It was also used to synthesise hierarchical porous MOF-based composites. The principle of this method is to form the solid around the droplets which are then removed by solvent evaporation, thereby leading to a porous material.^142^ Two kinds of emulsions were used: “water-in-oil” (W/O) emulsion in which water droplets are dispersed in an oil continuous phase or “oil-in-water” (O/W) emulsion in which oil droplets are dispersed in a water continuous phase. Highly interconnected porous MOFs/polymer monoliths and beads were prepared by using high internal phase emulsions (HIPEs) as templates.^149–151^ HIPE is an emulsion, characterised by a high volume of the internal droplet phase exceeding 74.05 v/v%.^142^ To enhance the thermodynamic stability of O/W emulsions, surfactants are generally added at concentrations allowing the formation of lyotropic mesophases. Foams termed polyHIPES can be formed through the polymerisation of the continuous phase and removal of the droplets in the dispersed phase. PolyHIPES can also be prepared by using Pickering emulsions that are emulsions stabilised by nanoparticles.^152^ The group of D. Bradshaw has shown that O/W Pickering emulsions stabilised by ZIF-8 NPs at the O/W interface could be used for the preparation of ZIF-8-polymer composite microcapsules.^153^ Such emulsions were formed by the application of shear force to a mixture of dodecane and an aqueous colloidal suspension of ZIF-8. This step was followed by the polymerisation of the dispersed phase at 65 °C since the oil phase contained the styrene and divinylbenzene monomers and initiator.^153^ A cross-linked polystyrene (PS) membrane was formed at the interface between the MOF NPs and dodecane due to the poor solubility of PS in dodecane (Fig. 9c). This phase separation of PS allowed the formation of hollow ZIF-8@PS microspheres in which the external surface is composed of ZIF-8 NPs embedded in the PS shell.^153^ According to TGA, the ZIF-8 content in these ZIF-8@PS microspheres is close to 20 wt%. This strategy was successfully extended to UiO-66(Zr) and MIL-101(Cr). It was shown that the capsule formation was driven by the PS phase separation that was dependent on the hydrophilic/hydrophobic balance of the MOF and the nature of the oil phase.^153^ According to SEM, the PS membrane presented a hierarchical and asymmetric porous structure that consisted of a disordered network of interconnected meso- and macropores covered by a denser polymer skin.^153^ The good efficiency of the ZIF-8@PS microcapsule for the capture of oil red dye resulted from a synergistic combination of a microporous MOF and a hierarchical porous polymer membrane.^153^ Then, D. Bradshaw and coworkers developed nanostructured and hierarchical porous ZIF-8/polyHIPES composites by inducing the polymerisation of the continuous phase in MOF-stabilized Pickering W/O emulsions (Fig. 9d).^154^ According to SEM and N_2_ porosimetry, this composite presented a multimodal porosity that comprised an array of macro- and mesopores in the PS matrix and micropores inherent in the ZIF-8 framework.^154^ Moreover, SEM images have shown that ZIF-8 NPs were homogeneously distributed in the PS and spatially organized in the internal surface of macropores. Therefore, the HIPE templating was shown to impart both hierarchical porosity and a nanostructuration of the MOF NPs in the polymer matrix.^154^ Composites were similarly prepared by using other MOFs (HKUST-1(Cu), MIL-101(Cr) and UiO-66(Zr)), thereby showing the versatility of this approach. These composites could be processed in different forms (spheres, monoliths, *etc*.) as a result of their good mechanical properties (Fig. 9d).^154^ Finally, the potential of the ZIF-8/PS composite as a heterogeneous catalyst was tested for the Knoevenagel condensation reaction. This composite presented a higher catalytic performance than the parent ZIF-8 NPs and this was mainly attributed to the high porosity of this material and the spatial arrangement of ZIF-8 NPs in the proximity of macropores.^154^ Macropores enhanced the diffusion rate of the analytes that were then converted by ZIF-8 NPs.

In the different examples described in this last section, different strategies were deployed to impart multiple porosities over lengths in the micro, meso to macroporous ranges in MOF composites. Such approaches took advantage of the possible association of MOFs with different complex fluids (colloidal suspensions, gels, foams, emulsions, *etc*) and the combined implementation of processing and/or drying techniques. Moreover, these strategies not only produce composites with a hierarchical porosity but also an adequate shaping for applications in catalysis, gas/liquid separation, *etc*.

## Conclusions and outlook

This perspective showed the diversity of synthetic strategies used to assemble MOFs with carbon-based materials. The development of a wide range of MOF-based composites took advantage of the structural and functional modularity of MOFs. In the first approach, the high and well-defined porosity of MOFs was exploited to encapsulate preformed carbon-based nanoentities (CQDs, fullerene, *etc.*). Different strategies were also explored to integrate polymers either in the framework or in the pores of MOFs. The design of polyMOFs relied on the integration of polymeric dicarboxylate ligands as part of the MOF lattice. In alternative approaches, the well-defined porous structure of MOFs was used as an environment for polymerisation reactions of a wide range of monomers. A high diversity of polymer-inside composites was thus prepared through the growth of polymers from pre-tethered active sites located at the MOF backbone. In numerous cases, the attractive interactions and close communication between both MOFs and polymers have allowed improvement of the chemical stability of MOFs and control of the MOF dynamics (flexibility) while imparting also novel functionalities including electron/proton conductivity, processability, mechanical or stimuli responsive properties among others. The design of composites was also extensively explored by restricting the assembly of carbon materials and (bio)polymers on the surface of MOF particles, with the aim of preserving the porosity of MOFs. However, numerous composites suffered from poor interfacial properties which led to different shortcomings such as high MOF aggregation, possible phase separation, structural defects or low porosity. Among the strategies envisaged to enhance the physico-chemical matching between MOFs and carbon materials, numerous functionalisation strategies of the MOF surface were concomitantly developed as described in this perspective article. The *in situ* formation of MOF in the presence of the carbon material was also shown to enhance the spatial distribution and structuration of MOF particles in the carbon-based matrix while also controlling the morphology of MOF particles through coordination modulation or template effects. One major difficulty in this area arises from the inherent complexity and heterogeneity of the surface chemistry of MOFs which is characterised by the presence of the organic linker, the IBU, vacant coordination sites, surface defects and coordinating solvent molecules. A computational approach based on molecular dynamics (MD) simulations was successfully developed by the group of G. Maurin to gain insight into the structure of MOF/polymer interfaces^57,155^ but the deployment of experimental tools was comparatively quite limited to different microscopies (SEM, TEM, and FIB-SEM tomography). Recently, the interactions of MOFs with different polymers within MMMs were described by combining solid-state NMR and MD simulations.^156^ It should be of interest to extend this approach to other MOF-based composites while exploring the combination of different experimental tools able to characterise the surface of MOFs or MOF-based composites such as X-ray photoelectron spectroscopy (XPS), dynamic nuclear polarization (DNP) surface enhanced NMR or multiple electron microscopies (HAADF-STEM, and STEM-EELS). In addition, the correlation between synthetic parameters (*i.e.* concentration of precursors, reaction time, temperature *etc.*) and the resulting microstructure/composition of MOF-based composites is often challenging to predict, especially for composites prepared through the bottle-around-ship strategy (*in situ* approach). As a result, the synthesis of MOF-based composites is typically achieved through trial and error rather than a systematic rational approach. However, “*in situ*” composites result generally from a complex mutual recognition between MOFs and carbon materials: the organic material can drive the nucleation and growth of the MOF while conversely, MOF crystal surfaces can direct the self-assembly processes and structuring of the carbon material. An in-depth understanding of such a complex interplay between both components would be very helpful to control and scale up the production of composites using reliable manufacturing protocols. Notably, significant progress has been made in understanding the formation mechanisms of MOFs through *in situ* and time-resolved studies, employing a range of experimental techniques (*i.e.* scattering methods, microscopies, and spectroscopies) as well as simulation techniques.^157^ A similar combination of advanced characterisation and simulation techniques in time-resolved and *in situ* studies would undoubtedly provide valuable insights into the formation of MOF/carbon material composites. Recently, the design of MOFs/carbon material composites with a hierarchical dimensionality, composition and porosity was also reported. However, the design of such composites is still in its infancy since these materials were often characterized by one type of structural hierarchy. Inspired by the amazing complex and hierarchical architecture of biominerals, a wide diversity of inorganic and organic materials (*i.e.* oxides, metals, calcium carbonate, hydrogels, *etc*.) with a hierarchy at multiple levels of porosity, composition, morphology and structure were developed for different applications (catalysis, separation, energy storage and conversion, life sciences, *etc.*).^121^ Therefore, it would be highly desirable to synthesise MOF/carbon materials with a high level of morphological complexity and multiple levels of structural hierarchy since this approach would be relevant to the design of novel types of catalyst supports, separation membranes, biomaterials in regenerative medicine, *etc.* This required also a multi-scale and advanced characterisation of such materials to evaluate the impact of such complex microstructures on their physico-chemical properties (diffusion kinetics, adsorption capacity, and catalytic activity) and performance for various applications. In particular, the pore size distribution, and connectivity of the micro-, meso and macroporosities of these hierarchically porous materials should be characterised by combining different experimental tools (N_2_ porosimetry, low-dose TEM, and ^129^Xe NMR). Another strategy to increase the complexity and functionality of composites relied on the assembly of MOFs with a variety of bioentities including cells, bacteria, yeasts or viruses.^158,159^ The nascent field of using MOFs for the preparation of living materials aims to exploit the unique properties of cells including stimuli-responsiveness, biocatalytic activity and self-repair. Although extensively explored with other materials, this biomimetic approach is still in its early stages of development with MOFs and MOFs have not yet reached the maturity of other immobilisation matrices such as silica or clays. Moreover, the assembly of MOFs with bioentities could be coupled with advanced processing strategies such as microfluidics. Given their operational characteristics, including simplicity, small sizes, short processing times, continuous operation, and improved transport properties, diverse microfluidic platforms (*i.e.* segmented microfluidics, digital microfluidic systems and synthesis in microdroplets) were recently used for the preparation of MOF particles, MOF hollow spheres, and MOF/polymer membranes as well as hierarchical yolk/shell MOFs/polymer composites.^160–162^ It could be envisioned to expand this approach for the production of a wider range of hierarchical MOF/(bio)organic material composites.

## Author contributions

K. D. and S. D. were involved in the literature search and the preparation of figures. S. D. compiled the list of references. N. S. conceptualized and wrote the perspective. Critical inputs were added by K. D., S. D. and E. D. All authors discussed the perspective. E. D. and N. S. reviewed and edited the final version of the perspective.

## Conflicts of interest

There are no conflicts to declare.

## Supplementary Material
